# Re-evaluation of dioxygenase gene phylogeny for the development and validation of a quantitative assay for environmental aromatic hydrocarbon degraders

**DOI:** 10.1093/femsec/fiv049

**Published:** 2015-05-05

**Authors:** Paola Meynet, Ian M. Head, David Werner, Russell J. Davenport

**Affiliations:** School of Civil Engineering and Geosciences, Newcastle University, NE1 7RU, England, UK

**Keywords:** dioxygenases, Rieske centre, quantitative PCR, phylogeny

## Abstract

Rieske non-heme iron oxygenases enzymes have been widely studied, as they catalyse essential reactions initiating the bacterial degradation of organic compounds, for instance aromatic hydrocarbons. The genes encoding these enzymes offer a potential target for studying aromatic hydrocarbon-degrading organisms in the environment. However, previously reported primer sets that target dioxygenase gene sequences or the common conserved Rieske centre of aromatics dioxygenases have limited specificity and/or target non-dioxygenase genes. In this work, an extensive database of dioxygenase α-subunit gene sequences was constructed, and primer sets targeting the conserved Rieske centre were developed. The high specificity of the primers was confirmed by polymerase chain reaction analysis, agarose gel electrophoresis and sequencing. Quantitative polymerase chain reaction (qPCR) assays were also developed and optimized, following MIQE guidelines (Minimum Information for Publication of Quantitative Real-Time PCR Experiments). Comparison of the qPCR quantification of dioxygenases in spiked sediment samples and in pure cultures demonstrated an underestimation of the Ct value, and the requirement for a correction factor at gene abundances below 10^8^ gene copies per g of sediment. Externally validated qPCR provides a valuable tool to monitor aromatic hydrocarbon degrader population abundances at contaminated sites.

## Abstract (precis)

Our study aimed to re-evaluate the phylogeny of Rieske non-heme iron dioxygenases using only retrieved primary nucleic acid sequences for the development of quantitative real-time PCR primers.

## Graphical abstract

**Graphical Abstract Figure.**
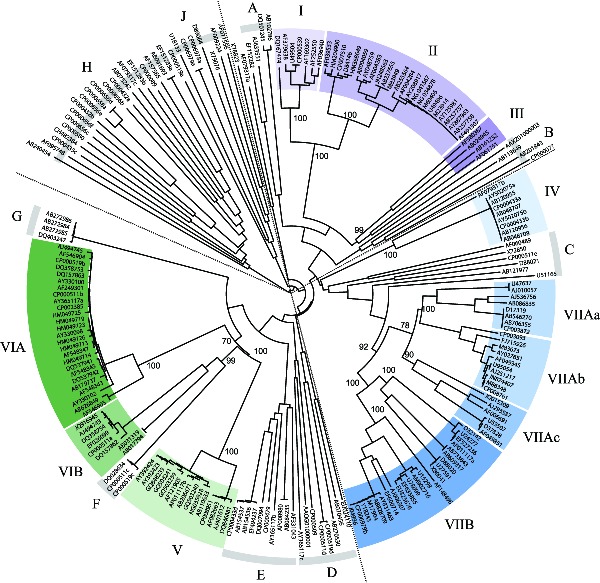
Our study aimed to re-evaluate the phylogeny of Rieske non-heme iron dioxygenases using only retrieved primary nucleic acid sequences for the development of quantitative real-time PCR primers.

## INTRODUCTION

Many aromatic hydrocarbons are EPA priority pollutants (USEPA 2001, 2010), due to their high residence time in the environment (Cerniglia 1992), and their toxic effects on biota, from microbes to humans, increase with their molecular size (Miller and Miller 1981; Fetzer 2003; Benigni and Bossa 2011). A broad range of phylogenetically diverse bacteria are able to catabolize aromatic hydrocarbons under both aerobic and anaerobic conditions or via cometabolism (Cerniglia 1992; Rockne and Strand 1998; Bamforth and Singleton 2005). Aerobic metabolism has received more attention, as it is more thermodynamically favourable (Bamforth and Singleton 2005). Dioxygenase enzymes and, in particular, the so-called Rieske non-heme iron oxygenases (RHOs) are a key family of enzymes, which initiate the aerobic degradation of aromatic hydrocarbons. The RHOs are multicomponent enzymes with an $\boldsymbol \alpha$_**n**_$\boldsymbol \beta$_**n**_ composition (Karlsson *et al.* 2003; Parales 2003; Ferraro, Gakhar and Ramaswamy 2005), where the α-subunit contains the catalytically relevant components and determines substrate specificity. The genes encoding these dioxygenases have been found in a wide range of aromatic-degrading bacteria (Habe and Omori 2003), either on plasmid or chromosomal DNA (Kweon *et al.* 2008). The classification schemes for RHOs proposed so far are mainly based on their occurrence in Gram-positive or Gram-negative bacteria (Cebron *et al.* 2008), substrate specificity (Wackett 2002; Iwai *et al.* 2011) or on each component of the RHO and their combined modular structure (Batie, Ballou and Correll 1991; Kweon *et al.* 2008). These classifications are generally constructed on the basis of homologies in the amino acid sequences of the components of each enzyme and their subunit structure, and included both mono- and dioxygenases. Recent attention has been focused on classification schemes based on the amino acid sequences of RHO α-subunits, due to their higher evolutionary plasticity compared to other RHO components and the fact that they contain the catalytically relevant metal centres (Wackett 2002; Vilchez-Vargas *et al.* 2013).

Iwai *et al.* (2011) have underlined how the current classifications underrepresent the diversity of dioxygenases in environmental samples, and, consequently, how the designed primers targeting the α-subunit fail to represent that diversity. Iwai's study called for a more comprehensive phylogenetic classification and a better approach to primer design, for which primer coverage, specificity and PCR product length should be considered. The limited effectiveness of the published primers is also reflected in the quantification of bacterial genes using molecular techniques, such as quantitative real-time PCR (qPCR), which has gained great popularity due to its conceptual and practical simplicity. High efficiency and accurate quantitative estimates can be obtained only if qPCR primers contain little degeneracy and amplify short fragment sizes (<250 bp) (Baldwin, Nakatsu and Nies 2003; Dionisi *et al.* 2004; Dorak 2006; Nyyssonen, Piskonen and Itavaara 2006; Cebron *et al.* 2008; Ding *et al.* 2010). However, the published primers employed in qPCR have often been designed to target RHOs genes from a limited range of bacterial taxa (Lloyd-Jones *et al.* 1999; Baldwin, Nakatsu and Nies 2003; DeBruyn, Chewning and Sayler 2007), others have not been specifically designed for qPCR but they have been adapted from general PCR in diversity studies (Laurie and Lloyd-Jones 2000; Yagi and Madsen 2009) that do not have ideal qPCR primers characteristics. In addition, most of those primers have been designed from phylogenetic analysis of RHO amino acid sequences, rarely from nucleic acid sequences (Baldwin, Nakatsu and Nies 2003), thereby leading to primers with high degeneracy related to codon translation. These approaches have resulted in primers that are either highly specific and selective (Lloyd-Jones *et al.* 1999; Wilson, Bakermans and Madsen 1999; Brezna, Khan and Cerniglia 2003) or highly degenerate (Ni Chadhain *et al.* 2006), and subsequently often not specific to aromatic dioxygenases. This is borne out in Iwai's analysis, where the degeneracies are high and perfect matches are low (see Fig. S1, Supporting Information, in Iwai *et al.* 2011).

In qPCR it is common to verify the ability of primers to amplify amplicons of the correct size, without actually assessing the accuracy of the quantification by calibration to some other measure (Ni Chadhain *et al.* 2006). In the present study, we set out to construct and re-evaluate the phylogeny for the genes encoding the α-subunit of the non-heme ring hydroxylating dioxygenases of aromatic hydrocarbon-degrading bacteria, with the intent to provide an updated phylogenetic classification of the RHOs. This was used as a framework with which to design a new series of highly specific primers targeting the conserved sequences of the catalytic Rieske centre of the different groups of aromatic dioxygenases, with the aim of developing a qPCR assay. These primers were optimized, validated against reference bacteria and used to develop standard curves. Finally, the accuracy of the quantification of targeted dioxygenases genes in environmental samples was evaluated by spiking sediment with known amounts of *Pseudomonas putida* DSM 8368, one of the aromatic degraders most commonly studied in engineered biodegradation systems (Sharma and Pathak 2014).

## MATERIAL AND METHODS

### Phylogenetic analyses

Reference nucleic acid sequences of the α-subunit (large subunit) of non-heme Rieske aromatic dioxygenases genes were retrieved from primary literature searches, and the GenBank (Benson *et al.* 2013) and EMBL-EBI (Pearson and Lipman 1988) databases (accessed in November 2014). The sequences were chosen by using the following criteria: (i) genes related to the Boolean search terms: ‘Rieske centre’, ‘α-subunit’, ‘aromatic ring-hydroxylating dioxygenase large subunit’; (ii) complete sequences; (iii) genes identified as dioxygenases (iv) sequences of genes referenced to primary literature citations; (v) environmental sources (sediment/soil/aquatic environments). The retrieved sequences were compared against the GenBank database (Benson *et al.* 2013) using the BLAST (Basic Local Alignment Search Tool) algorithm, and the homologous dioxygenases sequences, showing high identity (>85%), were added to the library. The final sequence library (for details, see Table S1 in Supporting Information) resulted in 209 nucleic acids of similar length (averagely 1359 ± 59 bp). ClustalX (Thompson *et al.* 1997) was used to perform pairwise and multiple alignments, followed by distance calculations using the neighbour-joining method. Evolutionary analyses were conducted in MEGA6 (Tamura *et al.* 2011), using the maximum composite likelihood model (Tamura, Nei and Kumar 2004) to estimate the evolutionary divergence between the sequences. A distance matrix (Table S2, in Supporting Information) was obtained where the values represented the dissimilarity for each pairwise comparison (phylogenetic diversity, PD). A phylogenetic tree was constructed by using the neighbour-joining method and visualized and manipulated using iTOL: Interactive Tree Of Life (Letunic and Bork 2007). The tree reliability was determined by 2000 bootstrap replications (for 95% reproducibility).

### Primer design

Primer sets were designed for 10 of the 20 clades identified in the phylogenetic tree, by aligning the corresponding nucleotide sequences of the large/α-subunits of dioxygenases targeting aromatic hydrocarbons and identifying the most conserved regions. Primers were manually designed by limiting their length to 16–20 oligonucleotides, annealing temperature between 55 and 70°C, GC content ≤ 60%, preventing self-complementary primers, and avoiding, where possible, degeneracies. The coverage for each respective clade was 100%. The specificity of primer sets to their selected clade was tested against the database constructed for phylogenetic analyses using Primrose software (Ashelford, Weightman and Fry 2002). The sequences of the chosen primers were also checked against the GenBank database to further confirm their specificity.

### Bacterial strains and cultures conditions

In order to test the specificity of the designed primers, a reference cultured bacterial strain was selected from every clade targeted by a primer set, and obtained from culture collections or private laboratories (see Table 1 for details). Liquid cultures were grown at the appropriate temperature (Table 1) in an orbital shaking incubator at 140 rpm. Solid media were prepared by the addition of 1% bacteriological agar (BDH Prolabo). Where needed (*Pandoraea pnomenusa* B-356, *Burkholderia xenovorans* LB400 and *Rhodococcus globerulus* P6), a carbon source was supplied to the media as biphenyl crystals, added as solid to the liquid medium (0.1% w/v) or a few crystals on the lids of inverted agar plates.

**Table 1.** Bacterial reference strains used in this study, their growth conditions and origin.

Clade	Bacteria strain	Growth conditions	Reference or source
**I & II**	*Pseudomonas putida* DSM 8368	Tryptone Soya Agar (Oxoid Cm131), 25°C	Evans, Fernley and Griffiths (1965)
**IV**	*Rhodococcus jostii.* RHA1*,1	Glucose yeast extract (Sambrook *et al.* 2001), 30°C	McLeod *et al.* (2006)
**V**	*Rhodococcus* sp. NCIMB12038	Nutrient agar (Oxoid CM3), 25°C	Boyd *et al.* (1997)
**VIA & VIB**	*Mycobacterium vanbaalenii* DSM 7251^T^ (PYR-1)	Brain heart infusion (Oxoid CM0375/0225), 30°C	Khan *et al.* (2002)
	*Mycobacterium* sp. SNP11*	Luria Bertani agar, 30°C	Pagnout *et al.* (2007)
**VIIAa**	*Pandorea pnomenusa* B-356*	M9 with biphenyl (≥98.0% SigmaAldrich) (Sambrook *et al.* 2001), 30°C	Gómez-Gil *et al.* (2007)
**VIIAb**	*Burkholderia xenovorans* LB400*	M9 with biphenyl (≥98.0% SigmaAldrich) (Sambrook *et al.* 2001), 30°C	Ferrer, Golyshin and Timmis (2003)
**VIIAc**	*Pseudomonas putida* 01G3*	Luria Bertani agar, 28°C	Jaouen *et al.* (2004)
**VIIB**	*Rhodococcus globerulus* P6*	M9 with biphenyl (≥98.0% SigmaAldrich) (Sambrook *et al.* 2001)	Asturias, Diaz and Timmis (1995)
	*Rhodococcus jostii.* RHA1*^,2^	Glucose yeast extract (Sambrook *et al.* 2001), 30°C	McLeod *et al.* (2006)

*Strains retrieved from private collections.

^1^Targeting etbA1/ebdA1/C genes

^2^Targeting bphA1 genes

### DNA extractions

DNA extractions from pure cultures were performed using FastDNA Spin Kit for Soil (MPBiomedicals, Santa Ana, CA, USA). The concentrations and purity of the DNA extracts were determined using a Nanodrop 1000 spectrophotometer (Thermo Scientific).

DNA extractions from sediment were carried out by modifying the FastDNA Spin Kit for Soil protocol, in order to prevent the coextraction of humic acids, clay minerals and other compounds that are known to inhibit molecular analysis. The modifications were taken from Griffiths *et al.* (2000), and consisted of the addition of 0.5 ml 0.12 M hexadecyltrimethylammonium bromide (CTAB) extraction buffer (pH 8) and 0.5 ml phenol:chloroform:isoamyl alcohol (25:24:1) to 0.5 g (wet weight) of sediment into a Lysing Matrix E tube of the FastDNA Spin Kit for Soil (MPBiomedicals, Santa Ana, CA, USA). The CTAB buffer was prepared by mixing equal volumes of 240 mM potassium phosphate buffer pH 8 (Sambrook, Fritsch and Maniatis 2001) with 10% (wt/vol) CTAB (Sigma-Aldrich, UK) in 0.7 M NaCl (Griffiths *et al.* 2000). Cell lysis was performed using a Fastprep Instrument (MP Biomedical, or Hybaid Ribolyser), at a speed of 5.5 m s^−1^ for 30 s. The aqueous phase containing nucleic acids was separated by centrifugation (14 000 × g) for 10 min, phenol was then removed from the aqueous phase by mixing with an equal volume of chloroform:isoamyl alcohol (24:1) and centrifuging at 14 000 × g for 5 min. The Binding Matrix Suspension, from the FastDNA Spin Kit for Soil, was used to bind the nucleic acid in the aqueous phase, before continuing as instructed by the manufacturer's protocol.

### PCR analysis

#### Annealing temperature optimization

The annealing temperature for each clade-specific primer set was optimized using genomic DNA extracted from the selected reference bacterial strain for the respective clade. A gradient of annealing temperatures, 52–70°C, was used. The PCR reactions were performed in a total volume of 25 μl, containing 10 μM of each primer, 0.2 μM of DNA template (or sterile molecular grade water, for the negative control) and 0.2 mM dNTPs, 1U BioTaq enzyme contained in the MegaMix Blue PCR master mix (Microzone Ltd, Haywards Heath, UK). The following thermocycler program was used for the amplifications: a denaturation cycle at 95°C for 1 min followed by 30 cycles of denaturation at 95°C for 30 s, 1 min at the annealing temperature (ranging from 52 to 69°C) and elongation at 72°C for 1 min, with a final elongation step at 72°C of 10 min. The PCR products were stored at −20°C until further use. To confirm the presence of the DNA in the sample, the purity of the PCR products and the amplification of the correct size gene fragments, DNA extractions and PCR products were routinely analysed using electrophoresis, run at 100 V for approximately 45 min, using 7 μl of product/extract on 1% agarose gels, containing 0.2 μg ml^−1^ of ethidium bromide (Sigma-Aldrich) DNA stain, and in 1 x Tris-acetate-EDTA buffer. Gels were visualized using a UV-P gel documentation system (Genetic Research Instrumentation, Dunmow, UK). The optimum annealing temperature was considered to be the highest temperature at which a strong band was observed.

#### Specificity

The specificity of each clade-specific primer set was checked by PCR against each reference organism selected to represent each clade (Fig. S1, Supporting Information) at the determined optimum annealing temperature. The bands were excised from the agarose gel, purified using a QIAquick PCR Purification Kit (Qiagen) and sequenced with the corresponding forward primer (3.2 pmol μes^−1^ μL^−1^) using the ABI prism Big Dye Terminator Cycle Sequencing Ready reaction kit and an ABI Prism 377 DNA sequencer (Applied Biosystems, USA). The obtained sequences were compared against the GenBank database (Benson *et al.* 2013) using the BLAST algorithm, in order to determine the closest matching sequence identity.

### Quantitative real-time PCR analysis

The quantitative real-time PCR methodology was developed in accordance with the MIQE ( *‘*Minimum Information for Publication of Quantitative real-time PCR Experiments’) guidelines, which describe the minimum information necessary for evaluating qPCR experiments in order to ensure its scientific integrity (Bustin *et al.* 2009).

The qPCR experiments were conducted on a BioRad CFX96 (Hercules, CA) with a C1000 thermal cycler iCycler and software version 3.0 (BioRad CFX Manager). qPCR reactions were performed in 10 μl mixtures containing 3 μl (to assure a concentration between 5 pg and 50 ng of genomic DNA, as per BioRad guidelines) of template DNA (or sterile molecular grade water for negative control), 15 pmol of each primer, with the SsoFast EvaGreen Supermix for the CFX96 (Bio-Rad Labs Ltd, Hemel Hempstead, UK). The following temperature profile was used in the amplifications: denaturation at 98°C (2 min), followed by 40 cycles of 2 s of denaturation at 98°C and 5 s at the primer-specific annealing temperature. The different annealing temperatures were tested using a temperature gradient on the qPCR instrument. At the end of the qPCR experiment, melt-curve analysis was performed: the qPCR products were subjected to a gradual increase of temperature (0.2°C temperature increments every 10 s) from 75 to 95°C, and the corresponding rate of change in SYBR Green fluorescence signal intensities was plotted as a function of temperature. Standard curves were constructed using plasmid clones of the target sequences (details in the following section), and generated every time a qPCR experiment was performed, in parallel with the amplification of test samples. All samples were run in triplicate, using the optimum qPCR conditions specifically determined for each primer set. The standard curves were graphically represented by plotting the Ct value (i.e. the cycle number at which the increase of fluorescence becomes exponential—threshold) versus the logarithm of the gene number (corresponding to the standard plasmid number).

### Standards for calibration of real-time PCR

Fresh PCR products of ring-hydroxylating α-subunit gene fragments from the reference strains were isolated by agarose gel electrophoresis, the corresponding bands excised and purified using a QIAquick PCR purification kit (QIAGEN, Crawley, UK). The purified products were then cloned into a pCR4 vector, following the TOPO TA cloning method according to the manufacturer's instructions (Invitrogen, Life Technologies, Paisley, UK). After plasmid purification (High Pure Plasmid Isolation Kit, Roche Ltd, Burgess Hill, UK), the plasmid DNA concentration was quantified using Nanodrop 1000 spectrophotometer (Thermo Scientific). The number of molecules (gene copies) of standard genes was calculated according to plasmid size (3973 bp) and insert length, assuming a molecular mass of 660 Da per base pair. Stock solutions of reference DNA standards were prepared at a concentration of 10^9^ copies of genes μl^−1^. For the calibration curves, DNA standards ranging from 10^9^ to 10^1^ target gene copies μl^−1^ were prepared by making a 10 times dilution series from the stock solutions of standard plasmid of 10^9^ target gene copies μl^−1^.

### Spiking experiment to validate qPCR assay

Fine-grained surface sediment grab samples were collected from the estuary of the river Coquet at Warkworth village (Northumberland, UK) and stored at +4°C until used. Detailed sediment characterization is reported in Siavalas *et al.* (2013). Autoclaved sediment was spiked with known concentrations (2.33 ± 0.07 × 10^9^ CFU ml^−1^, and 10-fold diluted to obtain concentrations of ∼ 2.33 × 10^8^ and 2.33 × 10^7^ CFU ml^−1^) of a pure culture of *P. putida* DSM 8368 containing *ndoB* gene (clade I–II) as follows. The range of concentration (10^7^−10^9^ CFU ml^−1^) was chosen to mimic the concentration range of *ndoB* genes previously found in sediments (Cebron *et al.* 2009).

The seed organism was grown in liquid media overnight, pelleted at 14 000 × g for 5 min, and washed once with sterile 10 mM phosphate buffer saline (pH 7.3, Oxoid, UK). The cells were repelleted and resuspended in phosphate buffer saline (PBS). The cells were then serially diluted in 10 mM PBS and 100 μl from each selected dilution (in the range of 10^7^–10^9^ CFU ml^−1^) was added to duplicate 0.5 g (wet weight) of autoclaved sediment sample. Autoclaved sediment samples were obtained by autoclaving twice at 121°C for 30 min. DNA was extracted in parallel from (i) autoclaved sediment, (ii) pure cultures of *P. putida* DSM8368 used to spike sediments and (iii) autoclaved sediment spiked with ∼ 10^7^, 10^8^ and 10^9^ CFU ml^−1^ *P. putida* DSM8368 pure culture, using the CTAB-modified FastDNA Spin Kit for Soil (Santa Ana, CA, USA) protocol (see ‘DNA extraction’ section for details). P1&2 primer set was used to qPCR quantify the *ndoB* genes in the DNA extracts. Each qPCR assay was performed at least twice, and all samples were measured in triplicate in each qPCR run, generating a minimum of six abundance measurements for each sample.

## RESULTS

### Phylogeny of dioxygenase $\boldsymbol \alpha$-subunits

**Figure 1.**
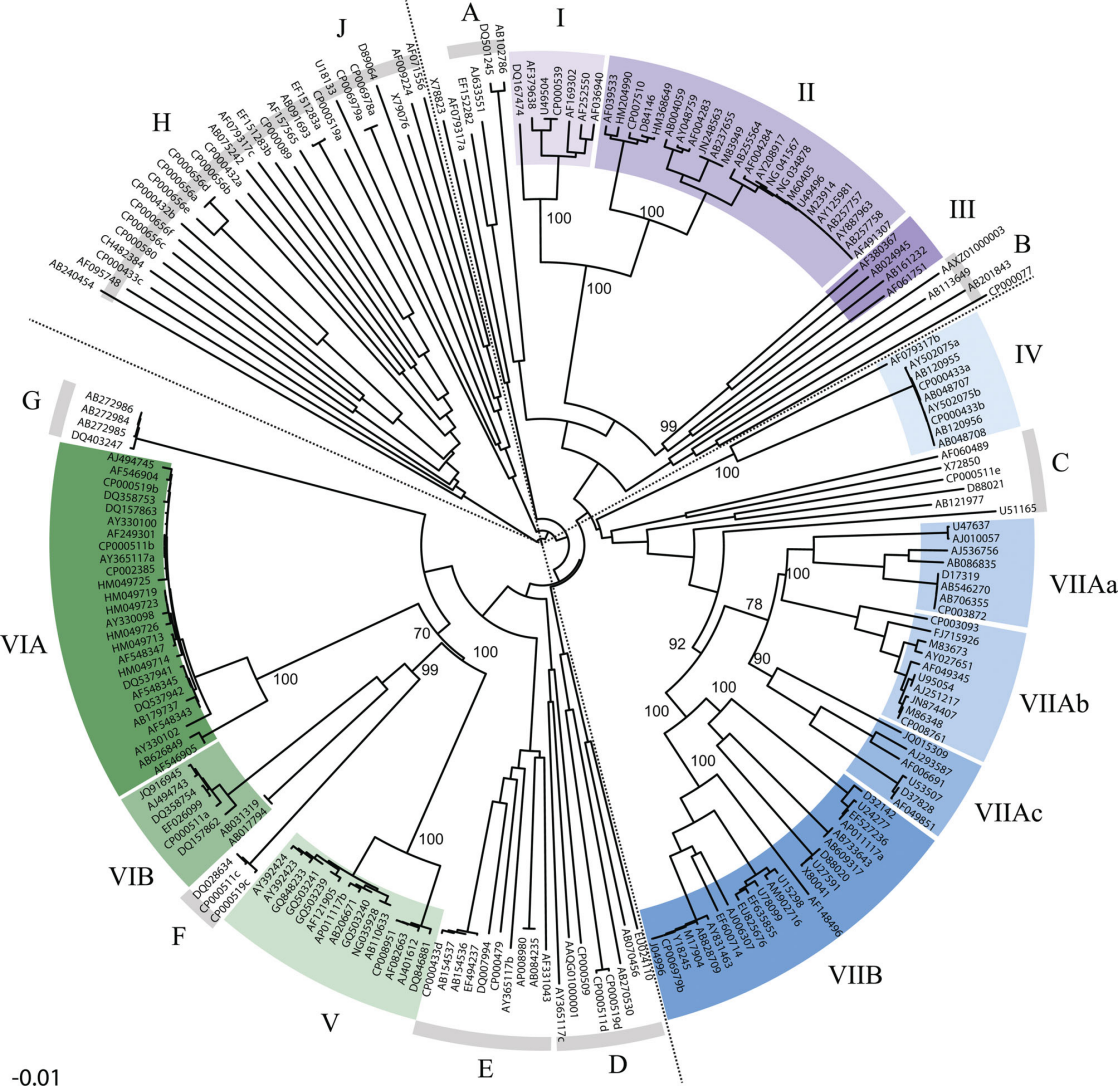
Phylogenetic neighbour-joining tree of nucleic acid sequences of the α-subunit (large subunit) gene of aromatic dioxygenases. The sequences are reported as GenBank accession number (see Table S1, in Supporting Information, for details on each sequence). The dotted lines define the four main lineages: lineage 1 (purple clades), composed of clades I (naphthalene dioxygenases), II (naphthalene dioxygenases), III (biphenyl dioxygenases), A (PAH dioxygenases), and B (dibenzofuran dioxygenases); lineage 2 (blue clades), composed of clades IV (alkylbenzene/aromatic-ring hydroxylation dioxygenases), VIIAa (biphenyl dioxygenases), VIIAb (biphenyl dioxygenases), VIIAc (alkylbenzene dioxygenases) and VIIB (toluene/biphenyl dioxygenases), and clade C (phenylproprionate/dioxin dioxygenases); lineage 3 (green clades), composed of clades D (extradiol/aromatic dioxygenases), E (phthalate dioxygenases), V (naphthalene dioxygenases), VIA (aromatic/PAHs dioxygenases) and VIB (phenanthrene/benzoate dioxygenases), F (ring-hydroxylating dioxygenases) and G (biphenyl dioxygenases); and lineage 4 (grey clades), composed of clades H (phenylproprionate/phthalate/aromatic dioxygenases) and J (benzoate/aromatic dioxygenases). See Table S1 in Supporting Information for specific gene names. The numbers at the nodes represent bootstrap values (%) for 2000 replicates. The scale represents the nucleotide substitution per site.

Four major lineages were identified in the phylogeny of 209 dioxygenase gene sequences from aromatic hydrocarbon-degrading bacteria (see Fig. 1). Lineage 1, lineage 2 and lineage 3 were dominated by previously classified (Gibson and Parales 2000; Wackett 2002; Iwai *et al.* 2011) PAH dioxygenases from Gram-negative bacteria (PAH-GN), toluene/biphenyl dioxygenases (T/B) and PAH dioxygenases from Gram-positive bacteria (PAH-GP) respectively (Fig. 1, and Table S1 in Supporting Information). Though several subclades displayed closely related genes from numerous organisms with coherent substrate specificity (average PD for all clades <0.29; degraders of naphthalene in subclades I–II and V; biphenyl in subclades VIIAa-b and VIIB, ethylbenzene in clade IV, PAHs in subclades VIA-B and VIIAc, F and G), our phylogeny revealed that several other clades contained more distantly related genes with less coherent substrate specificity and function (average PD for all clades >0.816; biphenyl, phenyl-like and aromatic degraders in subclades III, A–E, H–J; Fig. 1). In addition, the multiple dioxygenase gene system of *Rhodococcus* sp. RHA1 (Seto *et al.* 1995; Iwasaki *et al.* 2007) was well resolved: *etbA1* (sequence AB120955) and *ebdA1* (sequence AB120956) genes, which share identical nucleotide sequence except for a single nucleotide, were found in clade IV; while *bphA1* (sequence D32142) gene is found in clade VIIB, as it displays only 39.2% identity with the genes in clade IV (Seto *et al.* 1995; Iwasaki *et al.* 2007). Similarly, in lineage 3, the *Mycobacterium* PYR-1 dioxygenase gene evolutionary structure (Stingley, Khan and Cerniglia 2004) was represented by genes *nidA* (sequence AY365117) in clade VIA, *orf25* (AY365117c) in clade D and *phtAa* (AY365117b) in clade E.

### Primer design and verification

Clades I–II, and IV–VIIB were used to design sets of primers having few redundancies and high specificity, because of the high evolutionary relatedness and coherent substrate specificities that they encompassed. These clades contained the majority of the available dioxygenase sequences and included those bacteria that have been most commonly found in previous studies (Gibson and Parales 2000; Wackett 2002; Iwai *et al.* 2011). Clades I and II were considered sister groups (average PD = 0.16) and were covered by a single primer set. Due to their high divergence, it was not possible to design primers that adequately targeted clades III (PD > 0.50, Table S2 in Supporting Information), A–E, H, J (for all clades PD > 0.44), and clades F and G were considered insufficiently characterized (too low number of sequences) (Fig. 1 and Table S2 in Supporting Information). Previously published primer sets that target some of these sequences (part of PAH-GN, OT-I and -II in Iwai *et al.* 2011) contain a high number of degeneracies and are not specific for those clusters (Iwai *et al.* 2011). Among our 18 primers, only one contained three degenerate bases, three contained two degenerate bases and four contained one degenerate base, with the remaining eight being perfect match primers (Table 2). The primer GC content was a mean of 54% (44–65%) and all amplicon sizes were less than 300 base pairs (bp; 60–269 bp). The coverage of each primer set for selected clades was 100% for members of the respective target clade. Their high specificity to the target genes was confirmed by BLAST analysis. The primer sequences had no hits with genes other than the expected dioxygenase genes (data not shown).

**Table 2.** PCR primer sets developed in this work.

Primer	Position in the model organism	Sequence* 5^′^→3^′^	Expected amplicon size (bp)	GC%	Annealing temperature (°C)	Clade specificity	Main target genes
P1&2 F	331	AACGG**Y**GAACTGCA**R**AGC	269	44	66	I, II	*nahAc*, *pahAc*, *ndoB*
P1&2 R	619	CGTCCAACC**V**ACGTGGTC		61			
P4 F	188	CCGGAGACTTCCTGACGAC	116	63	66	IV	*etbA1*, *ebdA1*
P4 R	322	GCA**S**ACGAA**Y**CGACGGGT		55			
P5 F	337	TGGACCTACAGCAACACG	101	55	67	V	*narAa*, *nidA*
P5 R	461	CCAGCGAGCCGAAGATCA		61			
P6A F	189	CGAGTCGGAGTTGGCCAAG	159	63	69	VIA, VIB	*nidA*, *pdoA1*
P6A R	366	CTGTACACCCAGCCGTGGT		63			
P6B F	262	TGGCGAACTCGTGTCGGCAC	195	65	65	VIA, VIB	*pdoA2*, *phdA*
P6B R	476	CGTCCAG**R**CAACCGAA**D**A**Y**C		50			
P7Aa F	229	ACCTACATGGGCGAAGACC	103	58	66	VIIAa	*bphA1*
P7Aa R	350	GTA**R**GTGCAGGTGAAGGCCT		55			
P7Ab F	165	TGGTCGCTCTTGGCTGTTAC	198	55	66	VIIAb	*bphA*
P7Ab R	382	GCTTGCCGGCGATGTCGTA		63			
P7Ac F	220	TA**Y**CTGACCACCTACATGG	60	47	62	VIIAc	*ipbA1*, *ebdAa*, *cuma1*
P7Ac R	298	CC**R**CGATGCCGACATTG		59			
P7B F	349	CAC**B**TGCAGCTA**Y**CACG	181	53	62	IV, VIIB	*bphA1*, *todC1*, *ipbA1*, *bpdC1*
P7B R	549	CATGTGGTCCATGTAGAAC		47			

*Bases in bold correspond to degenerate bases in IUPAC codes (Y = T, C; R = G, A; V = G, C, A; S = G, C; D = G, A, T; B = G, T, C).

Primer specificity to a single clade was assessed by examining the PCR products amplified from the DNA of all the reference strains used for each clade (Fig. S1 in Supporting Information), using each primer set at its optimized annealing temperature. Each primer set successfully amplified an intense clear product of the expected size from the genomic DNA of the reference organism for each of the respective clades (Fig. S1 in Supporting Information), and the sequencing of the amplified DNA fragments confirmed that the correct target gene was amplified (see Table 3 in Supporting Information for details).

**Table 3.** Summary of the optimum annealing temperatures and relative standard curves for the designed primers in qPCR. Linear fits to experimental data were performed by the least-squares analysis.

		Standard curve *y = ax + b*			
Primers set	Annealing T (°C)	Slope (*a*)	Intercept (*b*)	% Efficiency (100 * [10^(−1/slope)^−1])	*R*^2^
P1&2	60	−3.19 ± 0.12	37.79 ± 0.60	105.6	0.9922
P4	60	−3.13 ± 0.08	41.67 ± 0.47	108.5	0.9949
P5	60	−3.45 ± 0.03	39.41 ± 0.17	94.8	0.9997
P6A	59	−3.19 ± 0.05	39.65 ± 0.21	105.6	0.9989
P6B	58	−3.39 ± 0.07	40.43 ± 0.38	97.1	0.9972
P7Aa	58	−3.13 ± 0.05	36.96 ± 0.27	108.4	0.9984
P7Ab	60	−3.09 ± 0.11	37.66 ± 0.69	110.9	0.9916
P7Ac	56	−3.48 ± 0.04	39.01 ± 0.20	93.8	0.9994
P7B	59	−3.15 ± 0.07	39.06 ± 0.41	107.9	0.9962

### Quantitative real-time PCR (qPCR) optimization

The optimal annealing temperatures for qPCR were assessed for the nine primer sets, by testing a range of temperatures above and below the optimum annealing temperature previously determined for general PCR reactions, using the reference strains (Table 3). For each amplicon, melt-curve analysis showed a single, characteristic peak for reactions conducted at the optimal melting temperature determined for each reference strain. The reaction products were also run on an agarose gel, confirming that a single amplicon was produced corresponding to the single peak in the melt curve (Fig. S2b in Supporting Information). The annealing temperature giving the lowest Ct values (or threshold cycle, corresponding to number of cycles at which the fluorescence signal becomes detectable) and the corresponding highest melt peak was considered optimal and matched the strongest bands seen in the agarose gel electrophoresis (Fig. S2a, Supporting Information).

Standard curves indicated that the amplification efficiency was high (Table 3), which in practice should be within the range of 90–110% (Dorak 2006), indicating that the PCR reaction had proceeded well and that the assays were sensitive (Dorak 2006). All standard curves were linear (*R*^2^ > 0.99) over nine orders of magnitude (10^9^–10^1^ gene copies μl^−1^) (Table 3).

### Quantification of aromatic degraders in sediment samples using external reference validation and correction

The feasibility to compare dioxygenase gene abundances in environmental samples using this methodology was previously reported elsewhere (Meynet *et al.* 2012). In the current work, we tested the accuracy of the developed qPCR assay by spiking sediment samples with known amounts of an external reference organism (*P. putida* DSM8368, containing *ndoB*) and quantifying *ndoB* genes using the corresponding primer set (P1&2).

**Figure 2.**
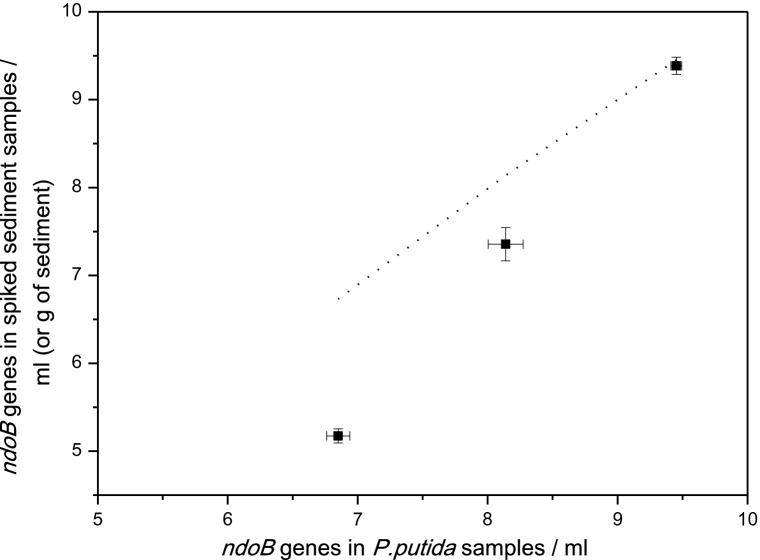
Cross-plot of the real-time PCR quantification (logarithmic scale) of duplicate DNA samples (extracted using CTAB method) of *P. putida* DSM8368 in concentrations in the order of 10^7^, 10^8^, 10^9^ CFU ml^−1^, as pure culture and in sediment matrix. Error bars indicate one standard deviation of two qPCR runs (each with triplicate qPCR reactions, i.e. *n* = 6) for each duplicate DNA samples (i.e. *n* = 12 for each point in the graph). The dotted line shows the theoretical recovery of *ndoB* genes in sediment samples when inhibiting effects are absent.

The qPCR abundance of *ndoB* genes (using P1&2 primer set) from duplicate DNA extracts of the *P. putida* DSM8368 pure cultures was used as a reference value for the evaluation of the DNA recovery and quantification sensitivity in the sediment samples. The *ndoB* qPCR abundances for the *P. putida* DSM8368 cultures used in the spikes matched the viable plate counts (least-square analysis, *r*^2^ = 0.997), which was initially used for determining the spike concentrations. When the DNA templates from sediment samples were undiluted or diluted 10×, the correspondent fluorescent signal was consistently lower than the one produced by the reference (*P. putida* DSM8368 DNA template), indicating the presence of PCR inhibitors in the qPCR reaction. Therefore, prior to qPCR, the DNA templates from sediment samples were diluted 100×, in order to dilute inhibitory materials that may remain after DNA purification. DNA templates from the *P. putida* DSM8368 pure culture were diluted in the same way for comparison. The abundance of *ndoB* genes measured in the CTAB-extracted DNA from either *P. putida* DSM8368 pure cultures and spiked sediments showed good reproducibility over the whole concentration range (10^7^–10^9^ gene copies/ml *P. putida* DSM8368 or g sediment) (Fig. 2). In DNA extracted from autoclaved sediment samples, target *ndoB* genes were undetectable by qPCR analysis (data not shown in Fig. 2), confirming the absence of any target dioxygenase genes after autoclaving. Melt-curve analysis confirmed the specificity of the assay for the chosen target gene, showing a single peak at the same melting temperature as the positive control (DNA from pure culture). At higher bacterial concentration (10^9^ gene copies per g of sediment), the abundance of *ndoB* genes measured in the DNA extracted from the spiked sediment matrix was statistically indistinguishable (*t*-test, *P* = 0.07) to the gene number detected in the reference samples *(P. putida* DSM8368 pure culture). At lower concentrations of spiked cells (in the range of 10^8^–10^7^ gene copies per g of sediment), the gene abundance measured in the spiked samples was statistically different (*t*-test, *P. putida* DSM8368 pure cultures versus spiked samples *P* < 0.00001 for both concentrations) compared to the reference samples, even though they maintained a linear relationship (*r*^2^ = 0.99979 with errors as weight). Thus, in the concentration range studied, the measured abundance in the spiked sediments is biased and needs to be corrected in order to truly represent the number of *ndoB* genes in the sample. Over the concentration range studied and for the sediment matrix used in this experiment, the real value of the concentration can be calculated using the following correction factor:

\begin{equation*}
LogC_{{\rm real}} = \frac{{LogC_{\rm m} + \left( {5.92 \pm 0.39} \right)}}{{\left( {1.62 \pm 0.05} \right)}},\end{equation*}

where *C*_m_ is the measured, *C*_real_ is the true concentration of *ndoB* genes in the sediment.

## DISCUSSION

Our extensive phylogenetic analysis of the α-subunits of dioxygenases identified 20 clades. It allowed us to design a set of highly specific primers for 10 clades, each of which had 100% coverage, because it was based on primary nucleic acid sequences. We validated the accuracy of one of the primer sets for qPCR of environmental samples by comparing a spiked sample with a reference control. This showed a very good accuracy at high cell numbers, but an order of magnitude error at lower cell numbers, suggesting that a correction factor based on the discrepancy with the external reference was required to accurately quantify gene abundances in environmental samples. Our classification identified four main lineages, in part similar to those previously reported in amino acid-derived phylogenies (Wackett 2002; Iwai *et al.* 2011), which grouped the dioxygenases depending on the substrates metabolized (Table S1 in Supporting Information). However, our analysis emphasized the distant relationship between some genes encoding aromatic dioxygenases for the oxidation of high molecular weight aromatic compounds, leading to some poorly defined diverse clades (clades A–J) within the main lineages. Furthermore, clades previously identified as other dioxygenases (OT-I and OT-II) (Iwai *et al.* 2011) were distributed throughout our tree (OT-I in subclades B, C, G and H; OT-II in subclades B and H. See Table S1 in Supporting Information). This suggests that more information is needed to better characterize those genes, in order to develop primer sets with higher specificity and good coverage than allowed by the highly degenerative primers currently available (Iwai *et al.* 2011). In our analysis, *Rhodococcus* sp. RHA1 ethylbenzene/C sequences, erroneously classified as PAH-GN and grouped with *Novosphingobium aromaticivorans* species in previous studies (Iwai *et al.* 2011), joined the toluene/biphenyl dioxygenases (T/B) clades in lineage 2, together with phenylproprionate/dioxin dioxygenases (subclade C), which in a previous study appeared to be distantly classified as other dioxygenases in the OT-I subclade (Iwai *et al.* 2011). Our phylogeny also resolved a more plausible structure for some multiple dioxygenase gene systems, such as *Rhodococcus* sp. RHA1 and *Mycobacterium* sp., which were previously underrepresented in other studies. This highlights how the multiple ring-hydroxylating dioxygenases large subunits, despite sharing conserved domains, do not have significant similarity in their nucleotide sequences, suggesting that they are distantly related (Stingley, Khan and Cerniglia 2004).

Phylogenies based on primary nucleic acid sequences may be better suited to designing primers targeting genes for either diversity or abundance studies, by enabling a reduction in excessive degeneracies generated from codon redundancy when translating amino acid sequences into nucleic acid sequences. We were therefore able to design highly specific qPCR primers with 100% coverage for 10 clades that had very high phylogenetic similarity (PD < 0.29, Table S2 in Supporting Information) and included the major and more common aromatic-degrading organisms isolated in soil and sediment samples (Daane *et al.* 2001; Hilyard *et al.* 2008). Their high specificity to the targeted gene was experimentally confirmed by PCR of target and closely related non-target genes from reference organisms followed by agarose gel analysis and sequencing of the PCR products. The PCR efficiency and sensitivity in our assay was improved by designing primers which generate short amplicons with high melting temperatures (Dorak 2006; Opel, Chung and McCord 2010). We have successfully applied our newly designed primer sets to compare dioxygenase gene abundances in environmental soil samples (Meynet *et al.* 2012). The use of primer sets that target the functionally important and evolutionarily conserved α-subunit, which contains the catalytic centre of very specific subsets of aromatic dioxygenases genes, decreases the probability of measuring abundance of other non-target dioxygenase gene populations. Baldwin, Nakatsu and Nies (2003) also adopted a similar approach, where naphthalene, toluene and biphenyl dioxygenases were the main dioxygenase groups studied. Their primer sets showed high specificity to the corresponding gene targets, but had low target coverage (Iwai *et al.* 2011) due to the limited number of reference sequences available at the time (i.e. three reference sequences for toluene dioxygenases). Almost all the primers in Baldwin's study produced amplicons over 400 bp, which can compromise the efficiency in qPCR assays (Dorak 2006).

Our results showed that qPCR maintained a very good accuracy in the quantification of high cell numbers (10^9^ genes number per g of sediment) in sediment samples, but for gene numbers ≤ 10^8^ the quantification was up to an order of magnitude lower than expected (spiked compared to reference control values), despite the good reproducibility of Ct values for both spiked and reference samples. The accuracy of quantifying gene abundances in environmental samples by qPCR is greatly influenced by DNA extraction and recovery efficiency, and PCR inhibition. The former is affected by the cell wall characteristics of the microorganisms containing the target gene, and the latter by the presence of humic acids. However, organic matter content of the environmental matrix (such as percentage of carbon and clay content) can also affect both DNA recovery efficiency and PCR inhibition (Zhou, Bruns and Tiedje 1996; van Doorn *et al.* 2009). This would account for the discrepancy observed between the spiked environmental sample and that of the control using a model reference organism: our protocol included strategies to account for extraction efficiency from a model organism with the target gene (by using an external control with the same cell wall characteristics) and to minimize inhibition from humic acids (the use of CTAB and dilutions). The use of an external control also provided a means by which to correct erroneous gene abundances.

Other studies have used a similar spiking approach (either employing external DNA or cells) to evaluate the DNA extraction recovery and/or the accuracy and sensitivity of PCR quantification assays in soil/sediment samples (Stults *et al.* 2001; Mumy and Findlay 2004; Coyne *et al.* 2005; Yun *et al.* 2006; Novinscak, Surette and Filion 2007; Trabelsi *et al.* 2009; Daniell *et al.* 2012). Few have used an external control with a model organism having the same cell wall characteristics as those that contain the target gene(s) (Stults *et al.* 2001). Stults *et al.* (2001) attributed inaccuracies in their TaqMan quantification to fluorescence quenching rather than to PCR inhibition or DNA extraction/recovery efficiency, which was assumed equal for all samples and gene concentrations. Our finding that the accuracy of qPCR quantification is concentration dependent (low target concentrations are more sensitive to PCR inhibition) is consistent with that of Van Doorn *et al.* (2009), who also demonstrated that PCR inhibition in environmental samples is higher with high organic matter content and depends on the soil composition. Furthermore, Zhou, Bruns and Tiedje (1996) demonstrated that although CTAB improved the removal of humic substances during DNA extraction, the DNA recovery could vary from 26 to 92% depending on soil organic matter content. We cannot rule out that the presence of organic matter may have influenced accuracy by affecting PCR inhibition and DNA recovery efficiency. Our results therefore emphasize the need of a correction system with which to calibrate qPCR assays of soil/sediment samples that takes into account these effects. The use of spiked environmental soil/sediment samples and controls using pure cultures of reference model organisms provided a valuable system for cross-calibrating and correcting qPCR abundance data. It is an approach that would benefit other similar studies.

## SUPPLEMENTARY DATA

Supplementary data is available at FEMSEC online.

Supplementary data is available at FEMSEC online
