# Supplementary material for: Re-evaluation of dioxygenase gene phylogeny for the development and validation of a quantitative assay for environmental aromatic hydrocarbon degraders
Source: FEMS Microbiol Ecol. 2015 May 5;91(6):fiv049. doi: 10.1093/femsec/fiv049 (PMC4462182; doi:10.1093/femsec/fiv049)
Supplement: Supplementary data is available at FEMSEC online [file Supporting_Information.docx]

Supporting Information

**Re-evaluation of dioxygenase gene phylogeny for the development and validation of a quantitative assay for environmental aromatic hydrocarbon degraders.**

Paola Meynet^[[1]](#footnote-1)^, Ian M. Head, David Werner, Russell J. Davenport

*School of Civil Engineering and Geosciences, Newcastle University, England, UK*

Content of the Supporting Information

|  |  | **Page** |
| --- | --- | --- |
| Table S1 | List of sequences used in this study | S2 |
| Text | Verification of primer-sets specificity | S17 |
| Figure S1 | Cross-reactions and specificity of the designed primer-sets | S18 |
| Table S2 | Estimate of the evolutionary divergence between the 120 nucleotide sequences considered in this study | Excel file |
| Table S3 | Summary of sequence identity of PCR products | S19 |
| Figure S2 | Optimisation of real-time PCR condition for primer P1&2. | S25 |
| Text | References | S26 |

**Table S1** List of sequences used in this study. The model organism used for each clade is reported in bold and blue. As comparison, the last column reports Iwai’s amino acid classification based on metabolised substrate. Sequences erroneously classified in Iwai et al as PAH-GN are marked with an asterisk (*).

| **GenBank accession n.** | **RHO enzyme system** | **Gene** | **Clade** | **Classification by metabolised substrate as in Iwai et al. (**[**Iwai *et al.*, 2011**](#_ENREF_1)**)** |
| --- | --- | --- | --- | --- |
| U49504 | 2-nitrotoluene dioxygenase (*Pseudomonas sp*. JS42) | ntdAc | I | PAH-GN |
| AF379638 | Naphthalene 1,2-dioxygenase (NDO) subfamily (*Comamonas sp.* JS765) | nbzAc | I | PAH-GN |
| AF169302 | Naphthalene 1,2-dioxygenase (NDO) subfamily (*Burkholderia cepacia* R34) | DntAc | I | PAH-GN |
| AF252550 | Polyaromatic hydrocarbon dioxygenase (*Comamonas testosteroni* H) | pahAc | I | PAH-GN |
| DQ167474 | Naphthalene 1,2 dioxygenase large oxygenase (*Polaromonas naphthalenivorans* CJ2) | nagAc | I | PAH-GN |
| AF036940 | Naphthalene 1,2 dioxygenase large oxygenase component (*Ralstonia* sp. U2 plasmid pWWU2) | nagAc | I | PAH-GN |
| CP000539 | Phenylpropionate dioxygenase alpha subunit (*Acidovorax* sp. JS42) | α subunit | I | PAH-GN |
| **M23914** | **Naphthalene dioxygenase (*Pseudomonas putida* NCIB9816)** | **ndoB** | **II** | **PAH-GN** |
| U49496 | Naphthalene dioxygenase (*Pseudomonas sp.* NCIB9816-4) | nahAc | II | PAH-GN |
| M60405 | Biphenyl dioxygenase (BPDO)-like subfamily (*Pseudomonas sp*.) | doxB | II | PAH-GN |
| AF004284 | Naphthalene dioxygenase (*Pseudomonas* *putida* ATCC 17484) | ndoC2 | II | PAH-GN |
| AB255564 | Naphthalene dioxygenase (*uncultured bacteria*) | nahAc | II | PAH-GN |
| AY048759 | Naphthalene dioxygenase (*Pseudomonas fluorescens*) | nahAc | II | PAH-GN |
| AF004283 | Naphthalene dioxygenase (*Pseudomonas fluorescens* ATCC 17483) | ndoC2 | II | PAH-GN |
| AB004059 | Polyaromatic hydrocarbon dioxygenase large subunit (*Pseudomonas putida* OUS82) | pahAc | II | PAH-GN |
| M83949 | Naphthalene dioxygenase (*Pseudomonas putida* G7) | nahAc | II | PAH-GN |
| AF039533 | Naphthalene dioxygenase (*Pseudomonas stutzeri* AN10) | nahAc | II | PAH-GN |
| D84146 | Naphthalene dioxygenase (*Pseudomonas aeruginosa* PaK1) | pahA3 | II | PAH-GN |
| NG_034878 | Naphthalene-1,2-dioxygenase (*Pseudomonas putida* plasmid NPL1) | nahA3 | II | - |
| NG_041567 | Naphthalene dioxygenase (*Pseudomonas putida* plasmid pAK5) | nahAc | II | - |
| AY208917 | Naphthalene-1,2-dioxygenase (*Pseudomonas putida* ND6 plasmid pND6-1) | nahAc | II | PAH-GN |
| AF491307 | Naphthalene-1,2-dioxygenase (*Pseudomonas putida* NCIB 9816-4 plasmid pDTG1) | nahAc | II | PAH-GN |
| AB257758 | Naphthalene-1,2-dioxygenase (Plasmid pFKY4 naphthalene degradation upper-pathway gene cluster) | nahAc | II | - |
| AY887963 | Naphthalene-1,2-dioxygenase (*Pseudomonas fluorescens* PC20 plasmid pNAH20) | nahAc | II | PAH-GN |
| AB257757 | Naphthalene-1,2-dioxygenase (Plasmid pFKY1) | nahAc | II | - |
| AY125981 | Naphthalene-1,2-dioxygenase (*Pseudomonas fluorescens* plasmid pLP6a) | nahAc | II | PAH-GN |
| AB237655 | Naphthalene-1,2-dioxygenase (*Pseudomonas putida* plasmid NAH7) | nahAc | II | PAH-GN |
| JN248563 | Naphthalene-1,2-dioxygenase (*Pseudomonas sp.* MC1 plasmid KOPRI126573) | nahAc | II | - |
| CP007510 | Naphthalene-1,2-dioxygenase (*Pseudomonas stutzeri* strain 19SMN4 plasmid pLIB119) | α subunit | II | - |
| HM204990 | Naphthalene dioxygenase (*Pseudomonas stutzeri* strain NJ) | nahAc | II | - |
| HM368649 | Naphthalene dioxygenase (*Pseudomonas* sp. N1) | pahA3 | II |  |
| AF380367 | Dioxygenase alpha subunit (*Burkholderia sp.* DBT1) | dbtAc | III | PAH-GN |
| AB024945 | 3,4-dihydroxyphenanthrene dioxygenase  (*Alcaligenes faecalis* AFK2) | phnAc | III | PAH-GN |
| AF061751 | Dioxygenase alpha subunit (*Burkholderia sartisoli* RP007) | phnAc | III | PAH-GN |
| AB161232 | Ring-hydroxylating dioxygenase (Sphingomonas sp. A4) | arhA1 | III | PAH-GN |
| **AB048707** | **Aromatic ring hydroxylation dioxygenase C (*Rhodococcus* sp. RHA1)** | **C** | **IV** | **PAH-GN*** |
| AB120955 | Ethylbenzene dioxygenase alpha subunit (*Rhodococcus* *jostii*. RHA1) | etbA1 | IV | PAH-GN* |
| AB048708 | Aromatic ring hydroxylation dioxygenase C2 (*Rhodococcus* *jostii* RHA1) | C2 | IV | PAH-GN* |
| AB120956 | Ethylbenzene dioxygenase alpha subunit (*Rhodococcus jostii* RHA1) | ebdA1 | IV | PAH-GN* |
| AY502075a | Alkylbenzene dioxygenase large subunit (*Rhodococcus* sp. DK17 plasmid pDK2) | akbA1b | IV | PAH-GN* |
| CP000433b | Ethylbenzene dioxygenase alpha subunit (*Rhodococcus* *jostii*. RHA1 plasmid pRHL2) | etbAa1 | IV | - |
| AY502075b | Alkylbenzene dioxygenase large subunit (*Rhodococcus* sp. DK17 plasmid pDK2) | akbA1a | IV | PAH-GN* |
| CP000433a | Ethylbenzene dioxygenase alpha subunit (*Rhodococcus* *jostii*. RHA1 plasmid pRHL2) | etbAa2 | IV | - |
| AF079317b | Large subunit aromatic oxygenase (Sphingomonas aromaticivorans DSM 12444 plasmid pNL1) | bphA1b | IV | - |
| **AF082663** | **Naphthalene dioxygenase large subunit (*Rhodococcus* sp. NCIMB12038)** | **narAa** | **V** | **PAH-GP** |
| AJ401612 | Putative cis-naphthalene 1,2-dioxygenase (*Rhodococcus* sp. 1BN) | narAa | V | PAH-GP |
| AY392424 | Naphthalene dioxygenase (*Rhodococcus* sp. P200) | narAa | V | PAH-GP |
| AY392423 | Naphthalene dioxygenase (*Rhodococcus* sp. P400) | narAa | V | PAH-GP |
| AB206671 | Naphthalene-inducible dioxygenase large subunit (*Rhodococcus opacus* TKN14) | nidA | V | PAH-GP |
| AF121905 | Aromatic dioxygenase (*Rhodococcus* sp. I24) | nidA | V | PAH-GP |
| DQ846881 | Naphthalene terminal dioxygenase (*Rhodococcus opacus* R7) | narAa | V | PAH-GP |
| AP011117b | Naphthalene dioxygenase large subunit (*Rhodococcus opacus* B4 plasmid pROB02 DNA) | nidA | V | - |
| GQ503241 | Naphthalene dioxygenase large subunit (*Rhodococcus* sp. B2-1) | narAa | V | - |
| GQ503240 | Naphthalene dioxygenase large subunit (*Rhodococcus* sp. B13) | narAa | V | - |
| GQ848233 | Naphthalene dioxygenase large subunit (*Gordonia sp.* CC-NAPH129-6) | narAa | V | - |
| NG035928 | Dihydroxy-phenylalanine dioxygenase terminal dioxygenase large subunit (*Rhodococcus opacus* plasmid pWK301) | dod | V | - |
| AB110633 | Terminal dioxygenase large subunit (*Rhodococcus opacus* plasmid pWK301) | dodA | V | - |
| GQ503239 | Naphthalene dioxygenase large subunit (*Rhodococcus* sp. DB11) | narAa | V | - |
| CP008951 | Naphthalene 1,2-dioxygenase (*Rhodococcus opacus* strain R7 plasmid pPDG4) | ndo | V | - |
| AF548345 | Aromatic dioxygenase (*Mycobacterium frederiksbergense* FAn9T) | nidA | VI A | PAH-GP |
| AB179737 | PAHs dioxygenase (*Mycobacterium sp.* MHP-1) | nidA | VI A | PAH-GP |
| DQ537941 | Dioxygenase large alpha subunit (*Mycobacterium pallens* czh-8) | nidA | VI A | PAH-GP |
| DQ537942 | Dioxygenase large alpha subunit (*Mycobacterium gilvum* czh-101) | nidA | VI A | PAH-GP |
| AF548343 | Dioxygenase large alpha subunit (*Mycobacterium gilvum* PYR-GCK) | nidA | VI A | PAH-GP |
| AY330098 | Aromatic dioxygenase (*Mycobacterium sp* JLS) | nidA | VI A | PAH-GP |
| AY330100 | Aromatic dioxygenase (*Mycobacterium sp* KMS) | nidA | VI A | PAH-GP |
| DQ157863 | Putative dioxygenase large subunit (*Mycobacterium* sp. CH-2) | nidA | VI A | PAH-GP |
| **AF249301** | **Aromatic dioxygenase (*Mycobacterium vanbaalenii* PYR-1)** | **nidA** | **VI A** | **PAH-GP** |
| AY365117a | Naphthalene inducible dioxygenase (*Mycobacterium* *vanbaalenii* strain PYR-1) | nidA | VI A | PAH-GP |
| DQ358753 | Putative dioxygenase large subunit (*Mycobacterium* sp. CH-1) | nidA | VI A | PAH-GP |
| AF548347 | Putative dioxygenase large subunit (*Mycobacterium gilvum* sp. BB1) | nidA | VI A | PAH-GP |
| AJ494745 | PAH ring-hydroxylating dioxygenase (*Mycobacterium* sp. 6PY1) | pdoA1 | VI A | PAH-GP |
| AY330102 | Aromatic dioxygenase (*Mycobacterium* MCS) | nidA | VI A | PAH-GP |
| AF546905 | Putative ring-hydroxylating dioxygenase (Mycobacterium sp. S65) | pdoA | VI A | PAH-GP |
| CP000511b | 3-phenylpropionate dioxygenase (*Mycobacterium* *vanbaalenii* PYR-1) | α subunit | VI A | PAH-GP |
| CP000519b | Ring hydroxylating dioxygenase (*Mycobacterium* sp. KMS plasmid pMKMS01) | α subunit | VI A | PAH-GP |
| CP002385 | Ring-hydroxylating dioxygenase (*Mycobacterium gilvum* Spyr1) | α subunit | VI A | - |
| AF546904 | Pyrene degradation gene cluster (*Mycobacterium* sp. S65) | nidA | VI A | PAH-GP |
| HM049726 | Dioxygenase large subunit (*Mycobacterium* sp. py146) | nidA | VI A | - |
| HM049719 | Dioxygenase large subunit (*Mycobacterium* sp. py137) | nidA | VI A | - |
| HM049723 | Dioxygenase large subunit (*Mycobacterium* sp. py142) | nidA | VI A | - |
| HM049713 | Dioxygenase large subunit (Bacterium py114) | nidA | VI A | - |
| HM049714 | Dioxygenase large subunit (Bacterium py120) | nidA | VI A | - |
| HM049725 | Dioxygenase large subunit (*Mycobacterium* sp. py145) | nidA | VI A | - |
| AB626849 | Ring-hydroxylating dioxygenase large subunit (*Mycobacterium* sp. NJS-P) | pdoA | VIA | - |
| AB017794 | Phenanthrene dioxygenase (*Nocardioides* sp KP7) | phdA | VI B | PAH-GP |
| AB031319 | Phenanthrene dioxygenase (*Nocardioides* sp KP7) | phdA | VI B | PAH-GP |
| **EF026099** | **Putative ring-hydroxylating dioxygenase (*Mycobacterium* sp. SNP11)** | **phdA** | **VI B** | **PAH-GP** |
| DQ358754 | Putative PAH ring-hydroxylating dioxygenase (*Mycobacterium* sp. CH-1) | pdoA2 | VI B | PAH-GP |
| AJ494743 | Putative PAH ring-hydroxylating dioxygenase (*Mycobacterium* sp. 6PY1) | pdoA2 | VI B | PAH-GP |
| CP000511a | Benzoate 1,2-dioxygenase, alpha subunit [*Mycobacterium vanbaalenii* PYR-1]. | α subunit | VI B | PAH-GP |
| DQ157862 | Putative PAH ring-hydroxylating dioxygenase (*Mycobacterium* sp. CH-) | pdoA2 | VI B | PAH-GP |
| JQ916945 | Pyrene dioxygenase alpha subunit (*Pseudomonas* sp. Jpyr-1 plasmid pJpyr-1) | pydA | VI B | - |
| AB086835 | Biphenyl dioxygenase large subunit (*Comamonas testosteroni* TK102) | bphA1 | VIIAa | T/B |
| AJ536756 | Biphenyl 2,3-dioxygenase (*Ralstonia oxalatica*) | bphA1 | VIIAa | T/B |
| D17319 | Biphenyl dioxygenase (*Pseudomonas sp*. KKS102) | bphA1 | VIIAa | T/B |
| AJ010057 | Biphenyl dioxygenase (*Burkholderia sp*. JB1) | bphA1 | VIIAa | T/B |
| **U47637** | **Biphenyl dioxygenase (*Pandoraea pnomenusa* strain B-356)** | **bphA** | **VIIAa** | **T/B** |
| AB546270 | Biphenyl dioxygenase (*Acidovorax* sp. KKS102) | bphA1 | VIIAa | - |
| AB706355 | Biphenyl dioxygenase alpha subunit (*Comamonas testosteroni*) | bphA1 | VIIAa | - |
| CP003872 | Phenylpropionate dioxygenase and related ring-hydroxylating dioxygenases (*Acidovorax* sp. KKS102) | HcaE | VIIAa | - |
| AJ251217 | Biphenyl dioxygenase (*Pseudomonas* sp. B4) | bphA1 | VIIAb | T/B |
| U95054 | Biphenyl dioxygenase (*Pseudomonas* sp. B4) | bphA1 | VIIAb | T/B |
| **M86348** | **Biphenyl dioxygenase (*Burkholderia xenovorans* LB400)** | **bphA** | **VIIAb** | **T/B** |
| AF049345 | Biphenyl dioxygenase (*Pseudomonas pseudoalcaligenes* KF707) | bphA1 | VIIAb | T/B |
| AY027651 | Biphenyl dioxygenase (*Pseudomonas sp.* Cam-1) | bphA | VIIAb | T/B |
| M83673 | Biphenyl dioxygenase (*Pseudomonas pseudoalcaligenes* KF707) | bphA1 | VIIAb | T/B |
| FJ715926 | Biphenyl dioxygenase (*Pseudomonas putida* B6-2) | bphA1 | VIIAb | - |
| CP003093 | Phenylpropionate dioxygenase and related ring-hydroxylating dioxygenases (*Pseudoxanthomonas spadix* BD-a59) | HcaE | VIIAb | - |
| JN874407 | Biphenyl 2,3-dioxygenase alpha subunit (*Cupriavidus* sp. SK-4) | bphA1 | VIIAb | - |
| CP008761 | Biphenylbdioxygenase alpha subunit (*Burkholderia xenovorans* LB400) | bphA | VIIAb |  |
| AF049851 | Ethylbenzene dioxygenase (*Pseudomonas fluorescens* CA-4) | edoA1 | VIIAc | T/B |
| D37828 | Cumene dioxygenase (*Pseudomonas fluorescens* IP01) | cumA1 | VIIAc | T/B |
| U53507 | Isopropylbenzene-2,3-dioxygenase (*Pseudomonas* JR1) | ipbA1 | VIIAc | T/B |
| AF006691 | Isopropylbenzene dioxygenase (*Pseudomonas putida* RE204) | ipbAa | VIIAc | T/B |
| **AJ293587** | **Alkylbenzene dioxygenase (*Pseudomonas putida* 01G3)** | **EbdAa** | **VIIAc** | **T/B** |
| JQ015309 | Biphenyl dioxygenase large subunit (*Alcanivorax* sp. HA03) | bphA1 | VIIAc | - |
| D88020 | Biphenyl dioxygenase (*Rhodococcus erythropolis* TA421) | bphA1 | VIIB | T/B |
| M17904 | Benzene dioxygenase (*Pseudomonas putida*) | α-subunit | VIIB | T/B |
| J04996 | Toluene dioxygenase (*Pseudomonas putida*) | todC1 | VIIB | T/B |
| Y18245 | p-cumate dioxygenase (*Pseudomonas putida* strain DOT-T1E) | CmtAb | VIIB | T/B |
| AY831463 | Large α-subunit (*Pseudomonas putida* strain GJ31) | cbzAa | VIIB | T/B |
| AJ006307 | Toluene dioxygenase (*Ralstonia* sp. JS705) | mcbAa | VIIB | T/B |
| U15298 | Chlorobenzene dioxygenase (*Pseudomonas putida* strain P51) | tcbAa | VIIB | T/B |
| U78099 | Chlorobenzene dioxygenase (*Burkholderia* sp PS12) | tecA1 | VIIB | T/B |
| AB828709 | Toluene dioxygenase (*Pseudomonas putida*) | todC1 | VIIB | - |
| CP006979b | Benzene 1,2-dioxygenase (*Pseudomonas monteilii* SB3101) | α-subunit | VIIB | - |
| EF600714 | Chlorobenzene dioxygenase large subunit (*Pandoraea pnomenusa*) | CbsAa | VIIB | T/B |
| EU825676 | Terminal dioxygenase alpha-subunit (*Bordetella* sp. IITR02) | tcbAa | VIIB | - |
| AM902716 | Phenylpropionate dioxygenase (*Bordetella petrii* DSM 12804) | HcaE | VIIB | - |
| EF635855 | Chlorobenzene terminal dioxygenase (*Pseudomonas nitroreducens* J5-1) | tcbAa | VIIB | T/B |
| AF148496 | Benzene/Benzoate 1,2-dioxygenase subunit alpha (*Pseudomonas putida* transposon Tn5542) | bedC1 | VIIB | T/B |
| U24277 | Isopropylbenzene 2,3-dioxygenase (*Rhodococcus erythropolis* BD2) | ipbA1 | VIIB | T/B |
| EF527236 | Toluene dioxygenase (*Rhodococcus* sp. L4) | terpA | VIIB | T/B |
| D32142 | Biphenyl dioxygenase (*Rhodococcus* sp. RHA1) | bphA1 | VIIB | T/B |
| **X80041** | **Biphenyl dioxygenase (*Rhodococcus globerulus* P6)** | **bphA1** | **VIIB** | **T/B** |
| U27591 | Biphenyl dioxygenase (*Rhodococcus* sp. M5) | bpdC1 | VIIB | T/B |
| AB609317 | Biphenyl 2,3-dioxygenase (*Janibacter* sp. TYM3221) | bphAa | VIIB | - |
| AB733643 | Biphenyl 2,3-dioxygenase (*Janibacter* sp. TYM3221) | bphAa | VIIB | - |
| AP011117a | Benzene dioxygenase large subunit (*Rhodococcus opacus* B4 plasmid pROB02) | bnzA1 | VIIB | T/B |
| DQ501245 | PAH dioxygenase iron sulfur protein large subunit ([*Cycloclasticus* sp. P1](http://www.ncbi.nlm.nih.gov/Taxonomy/Browser/wwwtax.cgi?id=385025)) | phnA1 | A | - |
| EF152282 | Biphenyl/naphthalene dioxygenase alpha subunit (S*phingobium yanoikuyae*) | bphA1f | A | PAH-GN |
| AJ633551 | Ring-hydroxylating dioxygenase alpha subunit (*Sphingomonas* sp. CHY-1) | phnA1a | A | PAH-GN |
| AB102786 | PAH dioxygenase (*Cycloclasticus* sp. A5) | phnA1b | A | PAH-GN |
| AB201843 | Dibenzofuran dioxygenase (*Paenibacillus* sp. YK5) | dbfA1 | B | OT-I |
| AAXZ01000003 | 3-phenylpropionate dioxygenase alpha subunit, (*Rhodobacteraceae bacterium* HTCC2150) | α-subunit | B | OT-I |
| AB113649 | Biphenyl dioxygenase (*Bacillus* sp. JF8) | bphA1 | B | PAH-GP |
| CP000077 | Aromatic ring dioxygenase subunit A (*Sulfolobus* *acidocaldarius* DSM 639) | α-subunit | B | OT-II |
| AF060489 | Carbazole dioxygenase (*Sphingomonas* sp. CB3) | carAa | C | T/B |
| X72850 | Dioxin dioxygenase (*Sphingomonas* sp.RW1) | dxnA1 | C | - |
| AB121977 | Anthranilate 1,2-dioxygenase (*Xanthobacter polyaromaticivorans* 127W) | dbdB | C | T/B |
| CP000511e | Phenylpropionate dioxygenase (*Mycobacterium vanbaalenii* DSM 7251 / PYR-1) | α-subunit | C | PAH-GP |
| D88021 | Terminal dioxygenase large subunit (*Rhodococcus erythropolis* TA421) | bphA1 | C | Fluorene |
| U51165 | Biphenyl dioxygenase (BPDO)-like subfamily (*Cycloclasticus oligotrophus* RB1) | XYLC1 | C | T/B |
| EU024110 | Fluorene/dibenzofuran angular dioxygenase large subunit (*Sphingomonas* sp. LB126) | flnA1 | D | OT-I |
| AB070456 | Extradiol dioxygenase for dibenzofurane degradation (*Rhodococcus* sp. YK2) | dbfA1YK2 | D | OT-I |
| AB270530 | Angular dioxygenase large subunit (*Sphingomonas* sp. KA1 plasmid pCAR3) | dbfA1 | D | Dibenzofuran |
| CP000519d | Ring hydroxylating dioxygenase (*Mycobacterium* sp. KMS plasmid pMKMS01) | α-subunit | D | PAH-GN |
| CP000511d | Ring hydroxylating dioxygenase (*Mycobacterium* *vanbaalenii* DSM 7251 / PYR-1) | α-subunit | D | PAH-GP |
| CP000509 | Aromatic-ring-hydroxylating dioxygenase, alpha subunit (*Nocardioides* sp. JS614). | α-subunit | D | PAH-GP |
| AAQG01000001 | Phenylpropionate dioxygenase and related ring-hydroxylating dioxygenases (*Sphingomonas* sp. SKA58) | HcaE | D | PAH-GP |
| AY365117c | Aromatic oxygenase large subunit (*Mycobacterium vanbaalenii* PYR-1) | α-subunit (orf25) | D | - |
| CP000479 | Phthalate dioxygenase large subunit (*Mycobacterium avium* 104) | α-subunit | E | OT-II |
| AB084235 | Oxygenase large subunit of phthalate dioxygenase (*Terrabacter* sp. DBF63) | phtA1 | E | PAH-GN |
| AY365117b | Phthalate dioxygenase large subunit (*Mycobacterium vanbaalenii* PYR-1) | phtAa | E | OT-II |
| CP000433d | Phthalate 3,4-dioxygenase (*Rhodococcus jostii* RHA1 plasmid pRHL2) | padAa | E | Phthalate |
| EF494237 | Phthalate 3,4-dioxygenase (*Rhodococcus* sp. DK17 plasmid pDK2) | ophA1 | E | Phthalate |
| AB154537 | Phthalate 3,4-dioxygenase (*Rhodococcus* sp. RHA1 plasmid pRHL2) | padAa2 | E | Phthalate |
| AB154536 | Phthalate 3,4-dioxygenase (*Rhodococcus* sp. RHA1 plasmid pRHL1) | padAa1 | E | Phthalate |
| DQ007994 | Phthalate dioxygenase (*Rhodococcus* sp. TFB) | phtAa | E | Phthalate |
| AP008980 | Phthalate 3,4-dioxygenase (*Terrabacter sp.* DBF63 plasmid pDBF1) | phtA1 | E | PAH-GN |
| AF331043 | Phthalate dioxygenase (*Arthrobacter keyseri* plasmid pRE1) | phtA1 | E | PAH-GN |
| DQ028634 | PAH ring-hydroxylating dioxygenase (*Mycobacterium vanbaalenii* PYR-1) | nidA3 | F | PAH-GP |
| CP000511c | Ring hydroxylating dioxygenase (*Mycobacterium vanbaalenii* PYR-1) | α-subunit | F | PAH-GP |
| CP000519c | Ring hydroxylating dioxygenase (*Mycobacterium* sp. KMS plasmid pMKMS01) | α-subunit | F | PAH-GP |
| AB272984 | Biphenyl dioxygenase (*Rhodococcus rhodochrous* K37) | bphA1 | G | OT-I |
| AB272986 | Biphenyl dioxygenase (*Rhodococcus* sp. HA99) | bphA1 | G | PAH-GN |
| AB272985 | Biphenyl dioxygenase (*Rhodococcus* *erythropolis* NCIMB 8147) | bphA1 | G | PAH-GN |
| DQ403247 | Biphenyl dioxygenase (*Rhodococcus* R04) | bphA1 | G | PAH-GN |
| AB091693 | Large subunit of oxygenase (*Sphingomonas* sp. P2) | ahdA1b | H | PAH-GN |
| AF157565 | Ring-hydroxylating dioxygenase (*Sphingopyxis macrogoltabida* strain TFA) | thnA1 | H | PAH-GN |
| CP000089 | Phenylpropionate dioxygenase (*Dechloromonas aromatic* RCB) | α-subunit | H | PAH-GN |
| CH482384 | Dioxygenase large alpha subunit (*Pseudomonas aeruginosa* 2192) | α-subunit | H | OT-I |
| CP000580 | Rieske-type ring hydroxylating dioxygenase alpha subunit (*Mycobacterium* sp. JLS) | α-subunit | H | OT-I |
| CP000656a | Phthalate 3,4-dioxygenase (*Mycobacterium gilvum* PYR-GCK) | α-subunit | H | OT-II |
| CP000656b | Phenylpropionate dioxygenase and related ring-hydroxylating dioxygenases (*Mycobacterium gilvum* PYR-GCK) | HcaE | H | OT-I |
| AB240454 | Large subunit of PAH-dioxygenase (*Sphingomonas* sp. A4) | arhA1 | H | PAH-GN |
| AF095748 | Phthalate dioxygenase (*Burkholderia cepacia* DBO1) | ophA2 | H | - |
| EF151283a | Ring hydroxylating dioxygenase alpha subunit (*Sphingobium yanoikuyae* strain B1) | bphA2b | H | PAH-GN |
| EF151283b | Ring hydroxylating dioxygenase alpha subunit (*Sphingobium yanoikuyae* strain B1) | bphA1a | H | PAH-GN |
| AF079317c | Large subunit naph/bph dioxygenase (*Sphingomonas aromaticivorans* plasmid pNL1) | bphA1f | H | PAH-GN |
| AB075242 | Dibenzofuran dioxygenase (*Terrabacter* sp. YK3 plasmid pYK3) | dfdA1 | H | dibenzofuran |
| CP000432a | Biphenyl 2,3-dioxygenase alpha subunit (*Rhodococcus jostii* RHA1 plasmid pRHL1) | bphAa | H | - |
| CP000656d | Ring hydroxylating dioxygenase (*Mycobacterium gilvum* PYR-GCK) | rdoA2 | H | PAH-GN |
| CP000656e | Phthalate 3,4-dioxygenase (*Mycobacterium gilvum* PYR-GCK) | phtAa | H | Phthalate |
| CP000656f | Ring hydroxylating dioxygenase (*Mycobacterium gilvum* PYR-GCK) | nidA | H | PAH-GP* |
| CP000656c | Ring hydroxylating dioxygenase (*Mycobacterium gilvum* PYR-GCK) | α-subunit | H | PAH-GN |
| CP000432b | Phthalate 3,4-dioxygenase (*Rhodococcus jostii* RHA1 plasmid pRHL1) | padAa | H | Phthalate |
| CP000433c | Terephthalate 1,2-dioxygenase (*Rhodococcus jostii* RHA1 plasmid pRHL2) | tpaAa | H | - |
| CP000519a | Ring hydroxylating dioxygenase (Mycobacterium sp. KMS plasmid pMKMS01) | α-subunit | J | PAH-GN |
| AF009224 | Benzoate 1,2-dioxygenase (*Acinetobacter* sp. ADP1) | benA | J | OT-I |
| AF071556 | Anthranilate dioxygenase (*Acinetobacter* sp. ADP1) | antA | J | - |
| X79076 | 2-halobenzoate 1,2-dioxygenase (*Burkholderia cepacia* 2CBS) | CbdA | J | Benzoate |
| D89064 | Carbazole 1,9a-dioxygenase (*Pseudomonas* sp. CA10) | carAa | J | - |
| CP006978a | p-cumate dioxygenase (*Pseudomonas monteilii* SB3078) | CmtAb | J | - |
| CP006979a | p-cumate dioxygenase (*Pseudomonas monteilii* SB3101) | CmtAb | J | - |
| U18133 | 3-chlorobenzoate-3,4-dioxygenase oxygenase (*Comamonas testosteroni* BR60) | cbaA | J | - |
| X78823 | Phenoxybenzoate dioxygenase (*Pseudomonas pseudoalcaligenes* POB310) | pobA | - | - |
| AF079317a | Aromatic oxygenase (*Sphingomonas aromaticivorans* DSM 12444 plasmid pNL1) | bphA1a | - | PAH-GN |

**Verification of primer-set specificity**

Sequencing of the amplified DNA fragments confirmed that the primer-sets amplified the correct target gene (Table S3). For 7 out of 9 (80%) of the primer-sets, no similar sized fragments were detected in the negative controls i.e. reference bacterial strains selected for other clades (Figure S1). The sequence of the amplified DNA fragments showed that P6A successfully amplified the *nidA* gene in both reference organisms for clade VIA and VIB), and BLAST analysis confirmed, respectively, 100% and 99% identity with *nidA* genes in *Mycobacterium* species. Similarly, the sequence analysis of fragments from P6B confirmed the amplification of the *pdoA* gene in both reference organisms for clade VIA and VIB, and BLAST analysis confirmed 100 and 98% identity with *pdoA* genes in *Mycobacterium* species. The coexistence of *nidA* and *pdoA* genes in both *Mycobacterium vanbaalenii* PYR-1 and SNP11 strains has been previously reported ([Stingley *et al.*, 2004](#_ENREF_5), [Pagnout *et al.*, 2007](#_ENREF_4)). Similarly, P7B successfully amplified *bphA* gene in both *Rhodococccus globerulus* P6 (sequence X80041) and *Rhodococcus* RHA1 (sequence D32142), and sequencing of the corresponding amplified DNA fragments confirmed this (Table S3, supporting information). It is well known that multiple RHOs genes are present in *Rhodococcus* sp. RHA1 ([Iwasaki *et al.*, 2006](#_ENREF_2)), which do not always show close identity in their nucleotide sequence. *Rhodococcus* sp. RHA1 harbours *etbA1* (sequence AB120955) and *ebdA1* (sequence AB120956), both in clade IV, which have a single nucleotide mismatch, but only 39.2% identity with *bphA1* from the same organism (([Iwasaki *et al.*, 2007](#_ENREF_3)), and in our phylogeny sequence D32142, clade VIIB; PD = 0.75 in Table S2). Primer-set P6B also amplified two larger fragments (400bp and 1,400 bp) in *Pseudomonas putida* 01G3 (carrying a clade VIIAc dioxygenase), which were failed to obtain sequences for. The full genome sequence of *Pseudomonas putida* 01G3 has not been determined. Together these results indicate that this strain may harbour chromosomal or plasmid sequences with similarity to the primers used, or the possible formation of concatemers. Altering the PCR conditions failed to eliminate these non-specific products obtained with primer P6B when used to amplify genes from *P. putida* 01G3. Interestingly, in qPCR assays primer set P6B produced a single peak in melting curve analysis with DNA from both pure cultures and environmental DNA samples (data not shown).

**Figure S1** Cross-reactions and specificity of the designed primer-sets evaluated by agarose gel electrophoresis. Markers correspond to 50-2,000-bp PCR marker (Sigma).

**Table S3** Summary of sequence identity of PCR products amplified with the primers designed in this study.

| **Primer set** | **Target organism (clade)** | **Closest relative (accession no.)** | **Identity (%)** |
| --- | --- | --- | --- |
| **P1&2** | *P.putida* DSM 8368 (I&II) | Pseudomonas putida plasmid pAK5 naphthalene dioxygenase reductase component (nahAa) (JQ922260.1) | 100% |
|  |  | Pseudomonas sp. 5K-VPa putative naphthalene dioxygenase (ndoB) gene ([JF520636.1\|](http://www.ncbi.nlm.nih.gov/nucleotide/335999238?report=genbank&log$=nuclalign&blast_rank=2&RID=V3H50Z8R015)) | 100% |
|  |  | Pseudomonas chlororaphis strain SY-02 plasmid pHL1 PAHs hydroxylase iron sulfur protein gene([HM623873.1\|](http://www.ncbi.nlm.nih.gov/nucleotide/304367818?report=genbank&log$=nuclalign&blast_rank=3&RID=V3H50Z8R015)) | 100% |
|  |  | Pseudomonas fluorescens strain PC20 plasmid pNAH20, complete sequence ([AY887963.3\|](http://www.ncbi.nlm.nih.gov/nucleotide/229424244?report=genbank&log$=nuclalign&blast_rank=4&RID=V3H50Z8R015)) | 100% |
|  |  | Uncultured bacterium clone B11 naphthalene dioxygenase alpha subunit (nahAc) gene ([EU660626.1\|](http://www.ncbi.nlm.nih.gov/nucleotide/195929460?report=genbank&log$=nuclalign&blast_rank=5&RID=V3H50Z8R015)) | 100% |
|  |  | Pseudomonas fluorescens plasmid pNAH20 naphthalene 1,2-dioxygenase iron sulfur protein component large subunit (nahAc) gene ([EF680322.1\|](http://www.ncbi.nlm.nih.gov/nucleotide/152143850?report=genbank&log$=nuclalign&blast_rank=21&RID=V3H50Z8R015)) | 100% |
|  |  |  |  |
| **P4** | *R. jostii.* RHA1 (IV) | Rhodococcus jostii RHA1 plasmid pRHL2, complete sequence ([CP000433.1\|](http://www.ncbi.nlm.nih.gov/nucleotide/110824911?report=genbank&log$=nuclalign&blast_rank=1&RID=VEHTYA6X015)) | 99% |
|  |  | Rhodococcus sp. DK17 plasmid pDK2, partial sequence ([AY502075.1\|](http://www.ncbi.nlm.nih.gov/nucleotide/40787187?report=genbank&log$=nuclalign&blast_rank=2&RID=VEHTYA6X015)) | 99% |
|  |  | Rhodococcus sp. RHA1 ebdA1, ebdA2, ebdA3, etbD2 genes for ethylbenzene dioxygenase alpha subunit, ethylbenzene dioxygenase beta subunit, ferredoxin, 2-hydroxt-6-oxohepta-2,4-dienoate hydrolase, complete cds ([AB120956.1\|](http://www.ncbi.nlm.nih.gov/nucleotide/35764425?report=genbank&log$=nuclalign&blast_rank=3&RID=VEHTYA6X015)) | 99% |
|  |  | Rhodococcus sp. RHA1 etbA1, etbA2, etbC, bphD, bphE2, bphF2 genes ([AB120955.1\|](http://www.ncbi.nlm.nih.gov/nucleotide/35764411?report=genbank&log$=nuclalign&blast_rank=4&RID=VEHTYA6X015)) | 99% |
|  |  | Rhodococcus sp. RHA1 gene for aromatic ring hydroxylation dioxygenase C2, complete cds ([AB048708.1\|](http://www.ncbi.nlm.nih.gov/nucleotide/15042005?report=genbank&log$=nuclalign&blast_rank=5&RID=VEHTYA6X015)) | 99% |
|  |  | Rhodococcus sp. RHA1 gene for aromatic ring hydroxylation dioxygenase C, complete cds ([AB048707.1\|](http://www.ncbi.nlm.nih.gov/nucleotide/15042003?report=genbank&log$=nuclalign&blast_rank=6&RID=VEHTYA6X015)) | 99% |
|  |  |  |  |
| **P5** | *Rhodococcus* sp*.* NCIMB12038 (V) | Rhodococcus opacus naphthalene degradation gene cluster, complete sequence; transposase (TnR7), rubredoxin (rub1), rubredoxin (rub2), trans-membrane proteins, and outer-membrane protein genes, complete cds; and unknown genes ([DQ846881.1\|](http://www.ncbi.nlm.nih.gov/nucleotide/110825894?report=genbank&log$=nuclalign&blast_rank=1&RID=VEJ2KTHE01R)) | 95% |
|  |  | Rhodococcus sp. NCIMB12038 naphthalene degradation gene cluster, complete sequence ([AF082663.3\|](http://www.ncbi.nlm.nih.gov/nucleotide/37683581?report=genbank&log$=nuclalign&blast_rank=2&RID=VEJ2KTHE01R)) | 95% |
|  |  | Rhodococcus sp. 1BN narA gene for putative cis-naphthalene 1,2-dioxygenase ([AJ401612.1\|](http://www.ncbi.nlm.nih.gov/nucleotide/10933938?report=genbank&log$=nuclalign&blast_rank=3&RID=VEJ2KTHE01R)) | 95% |
|  |  | Rhodococcus sp. CIR2 naphthalene degradation genes (rnoA1, rnoA2, rnoA3, rnoA4, rnoB), complete cds ([AB024936.1\|](http://www.ncbi.nlm.nih.gov/nucleotide/4586293?report=genbank&log$=nuclalign&blast_rank=4&RID=VEJ2KTHE01R)) | 95% |
|  |  | Rhodococcus sp. G10 naphthalene dioxygenase large subunit (narAa) and naphthalene dioxygenase small subunit (narAb) genes, partial cds ([GQ503238.1\|](http://www.ncbi.nlm.nih.gov/nucleotide/306490582?report=genbank&log$=nuclalign&blast_rank=5&RID=VEJ2KTHE01R)) | 91% |
|  |  | Rhodococcus sp. B2-1 naphthalene dioxygenase large subunit (narAa) and naphthalene dioxygenase small subunit (narAb) genes, partial cds ([GQ503241.1\|](http://www.ncbi.nlm.nih.gov/nucleotide/306490591?report=genbank&log$=nuclalign&blast_rank=6&RID=VEJ2KTHE01R)) | 91% |
|  |  | Rhodococcus sp. B13 naphthalene dioxygenase large subunit (narAa) and naphthalene dioxygenase small subunit (narAb) genes, partial cds ([GQ503240.1\|](http://www.ncbi.nlm.nih.gov/nucleotide/306490588?report=genbank&log$=nuclalign&blast_rank=7&RID=VEJ2KTHE01R)) | 91% |
|  |  | Gordonia sp. CC-NAPH129-6 rubredoxin (rub1), putative naphthalene degradation regulator protein (narR1), putative naphthalene degradation regulator protein (narR2), hypothetical protein, putative naphthalene dioxygenase large subunit (narAa), putative naphthalene dioxygenase small subunit (narAb), putative cis-naphthalene dihydrodiol dehydrogenase (narB), and putative aldolase (narC) genes, complete cds ([GQ848233.3\|](http://www.ncbi.nlm.nih.gov/nucleotide/325512092?report=genbank&log$=nuclalign&blast_rank=10&RID=VEJ2KTHE01R)) | 91% |
|  |  | Rhodococcus opacus B4 plasmid pROB02 DNA, naphthalene dioxygenase large subunit nidA ([AP011117.1\|](http://www.ncbi.nlm.nih.gov/nucleotide/226245746?report=genbank&log$=nuclalign&blast_rank=11&RID=VEJ2KTHE01R)) | 91% |
|  |  |  |  |
| **P6A** | *M. vanbaalenii* DSM 7251(PYR-1) (VIA) | Mycobacterium gilvum Spyr1, complete genome ([gb\|CP002385.1\|](http://www.ncbi.nlm.nih.gov/nucleotide/315259999?report=genbank&log$=nuclalign&blast_rank=1&RID=V3HY37KE01R)), [ring-hydroxylating dioxygenase, large terminal subunit](http://www.ncbi.nlm.nih.gov/nucleotide/315259999?report=gbwithparts&from=162204&to=163571&RID=V3HY37KE01R) | 100% |
|  |  | Mycobacterium tuberculosis strain SE12 dioxygenase large subunit (nidA) gene, partial cds ([gb\|GU586859.1\|](http://www.ncbi.nlm.nih.gov/nucleotide/291191428?report=genbank&log$=nuclalign&blast_rank=2&RID=V3HY37KE01R)) | 100% |
|  |  | Diaphorobacter sp. KOTLB clone 1 NidA (nidA) gene, partial cds ([gb\|FJ032196.1\|](http://www.ncbi.nlm.nih.gov/nucleotide/237825726?report=genbank&log$=nuclalign&blast_rank=3&RID=V3HY37KE01R)) | 100% |
|  |  | Mycobacterium gilvum PYR-GCK, complete genome ([gb\|CP000656.1\|](http://www.ncbi.nlm.nih.gov/nucleotide/145213092?report=genbank&log$=nuclalign&blast_rank=5&RID=V3HY37KE01R)) [ring hydroxylating dioxygenase, alpha subunit](http://www.ncbi.nlm.nih.gov/nucleotide/145213092?report=gbwithparts&from=595778&to=597154&RID=V3HY37KE01R) | 100% |
|  |  | Mycobacterium sp. JLS, complete genome ([gb\|CP000580.1\|](http://www.ncbi.nlm.nih.gov/nucleotide/126232413?report=genbank&log$=nuclalign&blast_rank=6&RID=V3HY37KE01R)), [ring hydroxylating dioxygenase, alpha subunit](http://www.ncbi.nlm.nih.gov/nucleotide/126232413?report=gbwithparts&from=2332127&to=2333503&RID=V3HY37KE01R) | 100% |
|  |  | Mycobacterium vanbaalenii PYR-1, complete genome ([gb\|CP000511.1\|](http://www.ncbi.nlm.nih.gov/nucleotide/119953846?report=genbank&log$=nuclalign&blast_rank=7&RID=V3HY37KE01R)),[ring hydroxylating dioxygenase, alpha subunit](http://www.ncbi.nlm.nih.gov/nucleotide/119953846?report=gbwithparts&from=530959&to=532326&RID=V3HY37KE01R) | 100% |
|  |  | Mycobacterium gilvum strain czh-101 dioxygenase large alpha subunit (nidA) gene, complete cds ([gb\|DQ537942.1\|](http://www.ncbi.nlm.nih.gov/nucleotide/108743910?report=genbank&log$=nuclalign&blast_rank=9&RID=V3HY37KE01R)) | 100% |
|  |  | Mycobacterium sp. S65 pyrene degradation gene cluster, partial sequence ([gb\|AF546904.1\|](http://www.ncbi.nlm.nih.gov/nucleotide/33333857?report=genbank&log$=nuclalign&blast_rank=13&RID=V3HY37KE01R)) | 100% |
|  |  | \|  \| Mycobacterium frederiksbergense strain FAn9T dioxygenase large alpha subunit (A) gene, complete cds (AF548345.1 ) \| \| --- \| --- \| | 100% |
| **P6A** | *Mycobacterium* sp. SNP11 (VIB) | Mycobacterium sp. py136 NidA (nidA) gene, partial cds ([gb\|HM049718.1\|](http://www.ncbi.nlm.nih.gov/nucleotide/296937352?report=genbank&log$=nuclalign&blast_rank=1&RID=V3VP54GN014)) | 99% |
|  |  | Bacterium py129 NidA (nidA) gene, partial cds ([gb\|HM049717.1\|](http://www.ncbi.nlm.nih.gov/nucleotide/296937350?report=genbank&log$=nuclalign&blast_rank=2&RID=V3VP54GN014)) | 99% |
|  |  | Pseudoxanthomonas sp. RN402 NidA (nidA) gene, partial cds ([gb\|FJ032197.1\|](http://www.ncbi.nlm.nih.gov/nucleotide/237825728?report=genbank&log$=nuclalign&blast_rank=6&RID=V3VP54GN014)) | 99% |
|  |  | Mycobacterium gilvum strain BB1 dioxygenase large alpha subunit (nidA) gene, complete cds [(AF548347.1\|](http://www.ncbi.nlm.nih.gov/nucleotide/26080262?report=genbank&log$=nuclalign&blast_rank=7&RID=V3VP54GN014)) | 99% |
|  |  | Mycobacterium gilvum strain czh-101 dioxygenase large alpha subunit (nidA) gene, complete cds ([DQ537942.1\|](http://www.ncbi.nlm.nih.gov/nucleotide/108743910?report=genbank&log$=nuclalign&blast_rank=27&RID=VEMNAW97014)) | 98% |
|  |  | Mycobacterium gilvum Spyr1, complete genome ([CP002385.1\|](http://www.ncbi.nlm.nih.gov/nucleotide/315259999?report=genbank&log$=nuclalign&blast_rank=8&RID=V3VP54GN014)), [ring-hydroxylating dioxygenase, large terminal subunit](http://www.ncbi.nlm.nih.gov/nucleotide/315259999?report=gbwithparts&from=162204&to=163571&RID=V3VP54GN014) | 98% |
|  |  |  |  |
| **P6B** | *Mycobacterium* sp. SNP11 (VIB) | Mycobacterium gilvum PYR-GCK, complete genome ([CP000656.1\|](http://www.ncbi.nlm.nih.gov/nucleotide/145213092?report=genbank&log$=nuclalign&blast_rank=2&RID=V3Y3GT0D01R)) | 98% |
|  |  | Mycobacterium vanbaalenii PYR-1, complete genome ([CP000511.1\|](http://www.ncbi.nlm.nih.gov/nucleotide/119953846?report=genbank&log$=nuclalign&blast_rank=3&RID=V3Y3GT0D01R)) | 98% |
|  |  | Mycobacterium sp. 6PY1 pdoF gene (partial), pdoA2 gene, pdoB2 gene and ORF1 DNA (AJ494743) | 98% |
|  |  | Mycobacterium sp. CH-1 putative dioxygenase, putative 3,4-dihydroxyphthalate decarboxylase, putative dihydrodiol dehydrogenase (phdE), putative extradiol dioxygenase (pdoF), putative PAH ring-hydroxylating dioxygenase large subunit 2 (pdoA2), and putative PAH ring-hydroxylating dioxygenase small subunit 2 (pdoB2) genes, complete cds; putative ferredoxin gene, partial cds; and unknown gene ([DQ358754.1\|](http://www.ncbi.nlm.nih.gov/nucleotide/90787427?report=genbank&log$=nuclalign&blast_rank=7&RID=V3Y3GT0D01R)) | 98% |
|  |  | Mycobacterium sp. SNP11 PAH degradation gene cluster, partial sequence ([EF026099.1\|](http://www.ncbi.nlm.nih.gov/nucleotide/116805444?report=genbank&log$=nuclalign&blast_rank=9&RID=V3Y3GT0D01R)) | 98% |
| **P6B** | *M.vanbaalenii* DSM 7251 (PYR-1) (VIA) | Mycobacterium vanbaalenii PYR-1 putative ring-hydroxylating dioxygenase (rdoA2) gene, partial cds ([EU872105.1\|](http://www.ncbi.nlm.nih.gov/nucleotide/195364351?report=genbank&log$=nuclalign&blast_rank=20&RID=V3WEM16D01R)) | 100% |
|  |  | Mycobacterium gilvum PYR-GCK, complete genome ([CP000656.1\|](http://www.ncbi.nlm.nih.gov/nucleotide/145213092?report=genbank&log$=nuclalign&blast_rank=1&RID=V3WEM16D01R)), [ring hydroxylating dioxygenase, alpha subunit](http://www.ncbi.nlm.nih.gov/nucleotide/145213092?report=gbwithparts&from=566003&to=567403&RID=V3WEM16D01R) | 98% |
|  |  | Mycobacterium vanbaalenii PYR-1, complete genome ([CP000511.1\|](http://www.ncbi.nlm.nih.gov/nucleotide/119953846?report=genbank&log$=nuclalign&blast_rank=2&RID=V3WEM16D01R)), benzoate 1,2-dioxygenase, alpha subunit | 98% |
|  |  | Mycobacterium sp. 6PY1 pdoF gene (partial), pdoA2 gene, pdoB2 gene and ORF1 DNA ([emb\|AJ494743.1\|](http://www.ncbi.nlm.nih.gov/nucleotide/27657407?report=genbank&log$=nuclalign&blast_rank=3&RID=V3WEM16D01R)) | 98% |
|  |  | Mycobacterium sp. JLS, complete genome ([CP000580.1](http://www.ncbi.nlm.nih.gov/nucleotide/126232413?report=genbank&log$=nuclalign&blast_rank=6&RID=V3WEM16D01R)), [ring hydroxylating dioxygenase, alpha subunit](http://www.ncbi.nlm.nih.gov/nucleotide/126232413?report=gbwithparts&from=1762812&to=1764212&RID=V3WEM16D01R) | 98% |
|  |  | Mycobacterium sp. CH-1 putative dioxygenase, putative 3,4-dihydroxyphthalate decarboxylase, putative dihydrodiol dehydrogenase (phdE), putative extradiol dioxygenase (pdoF), putative PAH ring-hydroxylating dioxygenase large subunit 2 (pdoA2), and putative PAH ring-hydroxylating dioxygenase small subunit 2 (pdoB2) genes, complete cds; putative ferredoxin gene, partial cds; and unknown gene ([DQ358754.1\|](http://www.ncbi.nlm.nih.gov/nucleotide/90787427?report=genbank&log$=nuclalign&blast_rank=7&RID=V3WEM16D01R)) | 98% |
|  |  | Mycobacterium sp. SNP11 PAH degradation gene cluster, partial sequence ([EF026099.1\|](http://www.ncbi.nlm.nih.gov/nucleotide/116805444?report=genbank&log$=nuclalign&blast_rank=9&RID=V3WEM16D01R)) | 98% |
| **P6B** | *P. putida* 01G3 (400bp fragment) (VIIAc) | No significant similarity found |  |
| **P6B** | *P. putida* 01G3 (1,500bp fragment) (VIIAc) | No significant similarity found |  |
|  |  |  |  |
| **P7Aa** | *Pandorea pnomenusa* B-356 (VIIAa) | Burkholderia sp. JB1 bphA1 gene ([AJ010057.1\|](http://www.ncbi.nlm.nih.gov/nucleotide/3413162?report=genbank&log$=nuclalign&blast_rank=1&RID=VBT5CNTV01R)) | 100% |
|  |  | Pandoraea pnomenusa strain B-356 biphenyl dioxygenase terminal oxygenase alpha subunit (bphA), biphenyl dioxygenase terminal oxygenase beta subunit (bphE), and biphenyl dioxygenase ferredoxin (bphF) genes, complete cds; 2,3-dihydro-2,3-dihydroxybiphenyl dehydrogenase (bphB) gene, partial cds; and unknown gene ([U47637.1\|CTU47637](http://www.ncbi.nlm.nih.gov/nucleotide/1245151?report=genbank&log$=nuclalign&blast_rank=2&RID=VBT5CNTV01R)) | 100% |
|  |  | Dyella ginsengisoli strain LA-4 biphenyl dioxygenase large subunit (bphA1), biphenyl dioxygenase small subunit (bphA2), membrane protein of biphenyl pathway, ferredoxin component of biphenyl dioxygenase (bphA3), ferredoxin reductase component of biphenyl dioxygenase (bphA4), dihydrodiol dehydrogenase (bphB), 2,3-dihydroxybiphenyl 1,2-dioxygenase (bphC), glutathione S-transferase (bphX0), 2-hydroxy-penta-2,4-dienoate hydratase (bphX1), meta-fission product hydrolase (mfphA), aldehyde dehydrogenase (bphX2), 4-hydroxy-2-oxovalerate aldolase (bphX3), and 2-hydroxy-6-oxo-6-phenylhexa-2,4-dienoic acid hydrolase (bphD) genes, complete cds ([EU258607.2\|](http://www.ncbi.nlm.nih.gov/nucleotide/198400352?report=genbank&log$=nuclalign&blast_rank=3&RID=VBT5CNTV01R)) | 89% |
|  |  | Dyella ginsengisoli biphenyl dioxygenase large subunit (bphA1) gene, partial cds ([EU391618.1\|](http://www.ncbi.nlm.nih.gov/nucleotide/166897666?report=genbank&log$=nuclalign&blast_rank=4&RID=VBT5CNTV01R)) | 89% |
|  |  |  |  |
| **P7Ab** | *Burkholderia xenovorans* LB400 (VIIAb) | Cupriavidus sp. SK4 BphA1-like (bphA1) gene, partial sequence (FJ416870.1) | 100% |
|  |  | Synthetic construct BphA-S151 (bphA) gene, complete cds (EF090939.1) | 100% |
|  |  | Burkholderia xenovorans LB400 chromosome 3, complete sequence, [biphenyl 2,3-dioxygenase alpha subunit (BphA1)](http://www.ncbi.nlm.nih.gov/nucleotide/91692731?report=gbwithparts&from=1252995&to=1254374&RID=V40PDJJ4014) ([CP000272.1\|](http://www.ncbi.nlm.nih.gov/nucleotide/91692731?report=genbank&log$=nuclalign&blast_rank=8&RID=V40PDJJ4014)) | 100% |
|  |  | Pseudomonas sp. Cam-1 bph operon, complete cds (AY027651.1) | 100% |
|  |  | Pseudomonas sp. B4 gene encoding putative transposase, ORF0 and ORF1 DNA, bphA1, bphA2, bphA3 and bphA4 genes (AJ251217.1) | 100% |
|  |  | Pseudomonas sp. LB400 biphenyl dioxygenase (bphA), biphenyl dioxygenase (bphE), biphenyl dioxygenase (bphF) and biphenyl dioxygenase (bphG)s, complete cds, and dihydrodiol dehydrogenase (bphB), partial cds (M86348.1) | 100% |
|  |  | Pseudomonas pseudoalcaligenes mutant biphenyl dioxygenase large subunit (bphA1) gene, complete cds (AF049345.1) | 100% |
|  |  | Pseudomonas sp. B4 biphenyl dioxygenase (bphA1) gene, complete cds (U95054.1) | 100% |
|  |  | P.pseudoalcaligenes dioxygenase (bphABC) gene cluster, complete cds (M83673.1) | 100% |
|  |  |  |  |
| **P7Ac** | *P. putida* 01G3 (VIIAc) | Pseudomonas putida ORF1 (partial), tnpA4 gene, tnpA3 gene, ebdAa gene, ebdAb gene, ebdAc gene, ebdAd gene, ebdB gene; ebdC gene and ebdE gene (partial), strain 01G3 (AJ293587.1) | 95% |
|  |  | Pseudomonas putida regulatory protein (ipbR), isopropylbenzene dioxygenase, iron-sulfur protein, large subunit (ipbAa), isopropylbenzene dioxygenase, iron-sulfur protein, small subunit (ipbAb), isopropylbenzene dioxygenase, ferredoxin subunit (ipbAc), isopropylbenzene dioxygenase, ferredoxin reductase subunit (ipbAd), cis-2,3-dihydroxy-2,3-dihydroisopropylbenzene dehydrogenase (ipbB), 3-isopropylcatechol dioxygenase (ipbC), 2-hydroxypenta-2,4-dienoate hydratase (ipbE), acetaldehyde dehydrogenase (acylating) (ipbG), 4-hydroxy-2-oxovalerate aldolase (ipbF), outer membrane protein (ipbH), and 2-hydroxy-6-oxo-7-methylocta-2,4-dienoate hydrolase (ipbD) genes, complete cds ([AF006691.1\|](http://www.ncbi.nlm.nih.gov/nucleotide/2822263?report=genbank&log$=nuclalign&blast_rank=2&RID=VEK7U89U01R)) | \|  \| 95% \| \| --- \| --- \| |
|  |  |  |  |
| **P7B** | *R. globerulus* P6 (VIIB) | R.globerulus genes bphA1-A4 ([X80041.1\|](http://www.ncbi.nlm.nih.gov/nucleotide/607171?report=genbank&log$=nuclalign&blast_rank=1&RID=V41VUFFS014)) | 99% |
|  |  | Rhodococcus erythropolis DNA for terminal dioxygenase, ferredoxin, ferredoxin reductase, complete cds ([D88020.1\|](http://www.ncbi.nlm.nih.gov/nucleotide/3059203?report=genbank&log$=nuclalign&blast_rank=2&RID=V4GKGE6X014)) | 99% |
|  |  | Rhodococcus sp. M5 terminal dioxygenase, large subunit (bpdC1), terminal dioxygenase, small subunit (bpdC2), ferredoxin (bpdB), ferrodoxin reductase (bpdA), 2,3-dihydroxy-4-phenylhexa-4,6-diene dehydrogenase (bpdD), and 2,3-dihydroxybiphenyl 1,2-dioxygenase (bpdE) genes, complete cds ([U27591.1\|](http://www.ncbi.nlm.nih.gov/nucleotide/927231?report=genbank&log$=nuclalign&blast_rank=3&RID=V4GKGE6X014)) | 99% |
| **P7B** | *R. jostii* RHA1 (IV) | [Rhodococcus sp. RHA1 biphenyl catabolic gene cluster (bphA1, bphA2, bphA3, bphC, bphB), complete cds](http://blast.ncbi.nlm.nih.gov/Blast.cgi" \l "alnHdr_510284" \o "Go to alignment for Rhodococcus sp. RHA1 biphenyl catabolic gene cluster (bphA1, bphA2, bphA3, bphC, bphB), complete cds) (D32142.1) | 100% |
|  |  | Uncultured bacterium clone BPA2.23 ring-hydroxylating dioxygenase (rhd) gene, partial cds ([EU146606.1\|](http://www.ncbi.nlm.nih.gov/nucleotide/157850589?report=genbank&log$=nuclalign&blast_rank=2&RID=VNPGKRDG01R)) | 100% |
|  |  | Rhodococcus jostii RHA1 plasmid pRHL1, complete sequence ([CP000432.1\|](http://www.ncbi.nlm.nih.gov/nucleotide/110823764?report=genbank&log$=nuclalign&blast_rank=44&RID=VNPGKRDG01R)) | 100% |
|  |  | Rhodococcus sp. L4 iron sulfur protein large subunit (terpA) gene, complete cds ([EF527236.1\|](http://www.ncbi.nlm.nih.gov/nucleotide/146262377?report=genbank&log$=nuclalign&blast_rank=42&RID=VNPGKRDG01R)) | 100% |
|  |  | Rhodococcus aetherivorans strain I24 dioxygenase large subunit gene, partial cds ([DQ813271.1\|](http://www.ncbi.nlm.nih.gov/nucleotide/112358842?report=genbank&log$=nuclalign&blast_rank=43&RID=VNPGKRDG01R)) | 100% |
|  |  | Rhodococcus aetherivorans strain I24 cosmid 2G11, partial sequence ([AF452376.1\|](http://www.ncbi.nlm.nih.gov/nucleotide/18150579?report=genbank&log$=nuclalign&blast_rank=45&RID=VNPGKRDG01R)) | 100% |
|  |  | Rhodococcus sp. Z6 biphenyl 2,3-dioxygenase alpha subunit (bphA1) gene, partial cds ([HM153082.1\|](http://www.ncbi.nlm.nih.gov/nucleotide/300247567?report=genbank&log$=nuclalign&blast_rank=94&RID=VNPGKRDG01R)) | 100% |
|  |  | Rhodococcus wratislaviensis strain IFP2016 IPB-dioxygenase ISP large subunit (ipbA1) gene, complete cds ([JX569344.1\|](http://www.ncbi.nlm.nih.gov/nucleotide/417072982?report=genbank&log$=nuclalign&blast_rank=48&RID=VNPGKRDG01R)) | 99% |
|  |  | Rhodococcus sp. ZWL3NT ntd gene cluster, complete sequence ([JX625147.1\|](http://www.ncbi.nlm.nih.gov/nucleotide/414146471?report=genbank&log$=nuclalign&blast_rank=49&RID=VNPGKRDG01R)) | 99% |

a)

b)

**Figure S2** Optimisation of real-time PCR condition for primer-set P1&2. a) Annealing temperature study: 60^0^C reaction gave the lowest C_t_ value and the highest peak in the melt curve analysis for this assay; b) Melt curve analysis: plot of the negative first derivative of the change in fluorescence as a function of temperature, where the peak indicates the melting temperature T_m_ of a specific PCR product. On the right, the corresponding agarose gel analysis of the real-time PCR product: lane 1, 50-2,000-bp PCR marker (Sigma); lane 2, real-time PCR product with P1&2 from the reaction with annealing T = 60^0^C.

**References**

Iwai S, Johnson TA, Chai BL, Hashsham SA & Tiedje JM (2011) Comparison of the Specificities and Efficacies of Primers for Aromatic Dioxygenase Gene Analysis of Environmental Samples. *Appl Environ Microbiol* **77**: 3551-3557.

Iwasaki T, Miyauchi K, Masai E & Fukuda M (2006) Multiple-subunit genes of the aromatic-ring-hydroxylating dioxygenase play an active role in biphenyl and polychlorinated biphenyl. degradation in Rhodococcus sp strain RHA1. *Appl Environ Microbiol* **72**: 5396-5402.

Iwasaki T, Takeda H, Miyauchi K, Yamada T, Masai E & Fukuda M (2007) Characterization of two biphenyl dioxygenases for biphenyl/PCB degradation in a PCB degrader, Rhodococcus sp strain RHA1. *Bioscience Biotechnology And Biochemistry* **71**: 993-1002.

Pagnout C, Frache G, Poupin P, Maunit B, Muller J-F & Férard J-F (2007) Isolation and characterization of a gene cluster involved in PAH degradation in Mycobacterium sp. strain SNP11: Expression in Mycobacterium smegmatis mc2155. *Research in Microbiology* **158**: 175-186.

Stingley RL, Khan AA & Cerniglia CE (2004) Molecular characterization of a phenanthrene degradation pathway in Mycobacterium vanbaalenii PYR-1. *Biochemical and Biophysical Research Communications* **322**: 133-146.

1. **Correspondence**: Paola Meynet, Environmental Engineering Group, School of Civil Engineering and Geosciences, Cassie Building, Newcastle University, Newcastle upon Tyne, NE1 7RU, UK. Tel. +44 (0)191 208 8479; fax: +44 (0)191 208 6502. E-mail: paola.meynet@ncl.ac.uk [↑](#footnote-ref-1)
